# *Bifidobacterium animalis* BD400 protects from collagen-induced arthritis through histidine metabolism

**DOI:** 10.3389/fimmu.2025.1518181

**Published:** 2025-01-22

**Authors:** Yang Yang, Qing Hong, Xuehong Zhang, Zhenmin Liu

**Affiliations:** ^1^ State Key Laboratory of Dairy Biotechnology, Shanghai Engineering Research Center of Dairy Biotechnology, Shanghai, China; ^2^ Dairy Research Institute, Bright Dairy & Food Co., Ltd., Shanghai, China; ^3^ State Key Laboratory of Microbial Metabolism, and School of Life Sciences and Biotechnology, Shanghai Jiao Tong University, Shanghai, China

**Keywords:** rheumatoid arthritis, *Bifidobacterium*, gut microbiota, metabolomics, early intervention

## Abstract

**Background:**

Rheumatoid arthritis (RA) is a common chronic and systemic autoimmune disease. Numerous clinical studies have indicated a correlation between alterations in gut microbiota and the onset and progression of RA. This research aims to restore intestinal microbiota to a healthy state through the oral administration of *Bifidobacterium* in the early stages with the goal of delaying the onset and progression of RA.

**Methods:**

Collagen-induced arthritis (CIA) rat model was constructed to assess the development of RA using arthritis clinical scores, paw thickness, pathological analysis of knee joint. The immune response was evaluated by determinating specific antibodies and cytokines in serum and synovial fluid. The expression of intestinal barrier protein was analyzed by qPCR to evaluate the intestinal barrier function. Alterations in gut microbiota and metabolites were assessed by 16S rDNA and non-targeted metabolomics.

**Results:**

The findings reveal that administering *Bifidobacterium animalis* BD400 orally led to a significant reduction in arthritis clinical scores and paw swelling thickness in CIA rats. Additionally, there was a decrease in osteo-facial fusion and calcified cartilage thickening in the knee joint. Furthermore, the oral administration of *B. animalis* BD400 resulted in the down-regulation of inflammatory factors TNF-α and collagenase MMP-13 in the knee joint. Levels of specific antibodies (anti-CII IgG, anti-CII IgG1, and anti-CII IgG2a) and cytokine IL-17A in serum, as well as cytokines (TNF-α and IL-1β) in the synovial fluid of *B. animalis* BD400-treated CIA rats, were significantly reduced (*p* < 0.05). The gene expression levels of intestinal barrier proteins (occludin-1, MUC-2, and ZO-1) showed a significant increase (*p* < 0.05) in *B. animalis* BD400-treated CIA rats. The oral administration of *B. animalis* BD400 altered the composition of intestinal microorganisms in CIA rats at the phylum and genus levels, particularly affecting the genus HT002. *B. animalis* BD400 alleviates RA by down-regulating 1-methyl-L-histidine and urocanate in the histidine metabolism, laying a foundation for the RA prevention.

**Conclusion:**

By affecting genus HT002 and histidine metabolism in the gut microbiota of CIA rats, *B. animalis* BD400 restored intestinal permeability, inhibited systemic inflammatory response, and ultimately slowed down the development of RA.

## 1 Introduction

Rheumatoid arthritis (RA) is an autoimmune disease characterized by persistent synovial inflammation, cartilage and bone damage, and potential disability (1). The global prevalence rates of RA range from 0.5% to 1.0% (1), with lower rates in China at 0.2% to 0.3% (2). RA is more common in females than in males (3). While the exact pathogenesis of RA remains unclear, certain risk factors such as family history, smoking, air pollution, periodontitis, and hormonal factors are associated with an increased likelihood of developing the disease (4). Studies have shown that RA patients exhibit distinct alterations in gut microbiota compared to healthy individuals (5, 6), which may contribute to systemic immune dysregulation (7, 8). Different strains of gut bacteria can have varying effects on immune system function, potentially influencing the development of RA (9). Disease-modifying anti-rheumatic drugs (DMARDs) are commonly used in RA treatment and may indirectly impact gut microbiota composition to modulate systemic immunity (9). Considering this therapeutic approach, there is interest in exploring whether early intervention targeting gut microbiota, such as probiotics, could potentially prevent the onset of RA.

Probiotics are defined as “live microorganisms that, when administered in adequate amounts, confer a health benefit on the host” (10). Probiotics can inhibit the growth of pathogenic bacteria by competing for nutrition and colonization sites. Meanwhile, metabolites of probiotics can strengthen the intestinal barrier and modulate the host immune responses (11). Three strains of *Lactobacillus acidophilus*, *Lactobacillus casei*, *Bifidobacterium bifidum* were provided for RA patients in a randomized, double-blind, placebo-controlled trial (12). After 8 weeks, RA patients receiving these probiotics showed improvement in Disease Activity Score of 28 joints (DAS-28) from -0.3 ± 0.4 to -0.1 ± 0.4. Furthermore, insulin levels, homeostatic model assessment-B cell function (HOMA-B), and serum high-sensitivity C-reactive protein (hs-CRP) concentrations decreased significantly, along with improvements in total and low-density lipoprotein-cholesterol levels (12). Given that most studies involve mixed strains of probiotics, the specific effects of a single strain, such as *Bifidobacterium*, remain unclear. While *Bifidobacterium* is known for its health benefits, further research is needed to determine if a single strain can replicate the effects of mixed probiotics. In this study, *B. animalis* BD400 is stored in the lactic acid bacteria species resource bank of the State Key Laboratory of Dairy Biotechnology. Compared with other *Bifidobacterium*, the *B. animalis* have good acid resistance, bile salt resistance and oxygen resistance, and have good survival ability in the human gastrointestinal tract. But the biological function of *B. animalis* BD400 is an unexplored area.

This study aims to protect from RA by taking *B. animalis* BD400 orally at an early stage to restore the balance of the unbalanced gut microbiota. Clinical scores for arthritis and paw swelling thickness were used to assess arthritis symptoms, while bone damage severity was determined through knee joint pathology and staining in rats. Immune response was evaluated by measuring specific antibodies and cytokines in serum and synovial fluid. The expression of intestinal barrier proteins was analyzed using qPCR to assess intestinal barrier damage. Changes in gut microbiota composition and diversity were assessed through 16S high-throughput sequencing, and metabolomics was used to analyze metabolite production and associated pathways in rat feces. The findings of this study offer valuable insights for the potential use of probiotics in the prevent of RA in daily life.

## 2 Materials and methods

### 2.1 Materials

Bovine type II collagen solution, complete Freund`s adjuvant and incomplete Freund`s adjuvant were purchased from Chondrex (Redmon, WA, USA). Methotrexate (MTX) was purchased from Shanghai Yuanye Bio-Technology Co. (Ltd, Shanghai, China). Anti-TNF-α antibody and anti-MMP-13 antibody were purchased from Abcam (Cambridge, United Kingdom). Total CII-IgG ELISA assay kit, CII-IgG1 ELISA assay kit, CII-IgG2a ELISA assay kit and CII-IgG2b ELISA assay kit were purchased from Shanghai MuLuan Biological Technology Co. (Ltd, Shanghai, China). Rat TNF-α ELISA assay kit, rat IL-1β ELISA assay kit and rat IL-17A ELISA assay kit were purchased from Hangzhou Lianke Biotechnology Co. (LTD, Hangzhou, China).

### 2.2 Bacterial strain

All the probiotics, listed in **Table 1**, were deposited at State Key Laboratory of Dairy Biotechnology. The twenty strains were cultured in DeManRogosa-Sharpe (MRS) medium at 37°C overnight, and were harvested by centrifugation at 5000g for 10 min at 4°C. The bacterial precipitate was washed twice with saline. The concentration of each strain was re-suspended at 5 × 10^8^ CFU/mL in saline, and stored at –80°C prior to use. The viability of all the probiotics suspensions was measured by colony counting before daily oral administration.

**Table 1:** Probiotics used in this study.

Group	Strains	Origin
*Bifidobacterium*	*B. longum* BD3150	Tibet feces
	*B. animalis* BD400	Granted
	*B. longum* BD6256 (PP4)	Long-lived people feces
	*B. bifidum* BD5348	Healthy human (1-3 years old)
	*B. breve* BB12	Granted

### 2.3 Animals and the experiment design

Female Wistar rats (7-8 week-old) (Shanghai SLAC Laboratory Animal Ltd, Shanghai, China) were allowed to acclimatize for seven days before experimentation. The rats were given free access to food and water specific pathogen-free (SPF) standard laboratory conditions at 25 ± 2°C and humidity of 50% ± 5%, with a 12 h light-dark cycle. This study was carried out in accordance with the Regulations for the Administration of Affairs Concerning Experimental Animals in China. Ethical approval for this study was obtained from Animal Ethics Committee of Shanghai Zhanyuan Biological Technology Co., LTD (approval No. 202306261).

### 2.4 Collagen-induced arthritis and treatment

**Figure 1:**
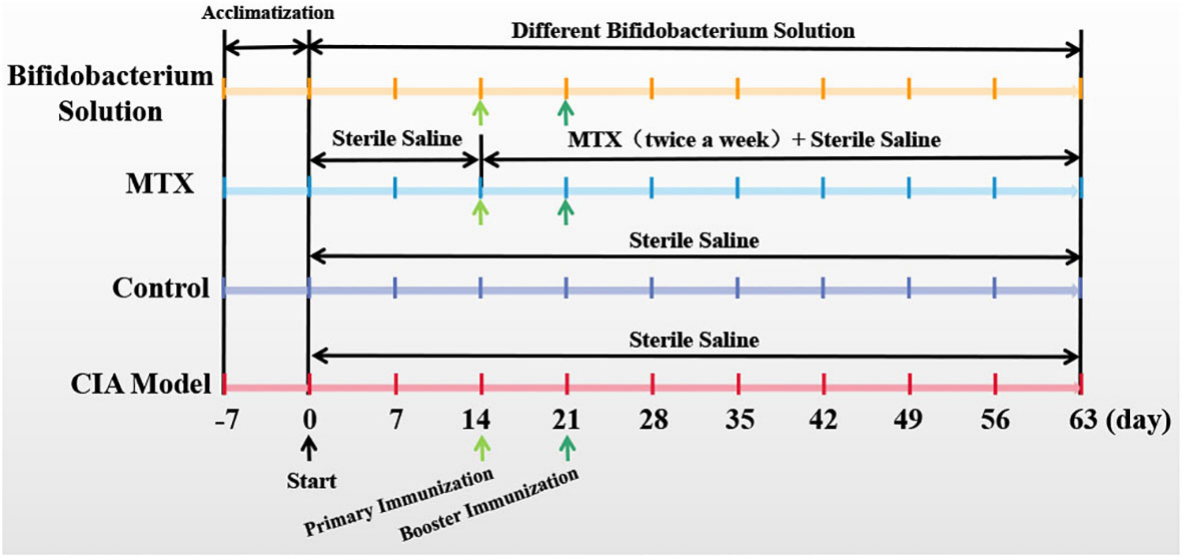
Experimental schedule.

The collagen-induced arthritis (CIA) animal model is the most commonly studied autoimmune model of rheumatoid arthritis. CIA was induced and assessed according to the previous method (13). Briefly, bovine type II collagen solution (CII, Chondrex, Redmon, WA, USA) were emulsified with complete Freund’s adjuvant (CFA, Chondrex, Redmon, WA, USA) at a ratio of 1:1, and 150 ml emulsion were injected subcutaneously into the tail root of each rat for multi-point immunization at day 1. After seven days, bovine type II collagen solution (CII, Chondrex, Redmon, WA, USA) were emulsified with incomplete Freund’s adjuvant (IFA, Chondrex, Redmon, WA, USA) at a ratio of 1:1, and 150 ml emulsion were injected into the tail root of each rat for a booster immunization. The control rats were subcutaneously injected with 150 ml sterile saline. The experimental rats were subcutaneously injected with same volume of probiotic liquid. The rats were sacrificed on the 63rd day by means of excessive anesthesia with a dose of 0.2 ml 3% pentobarbital sodium per 100 g of rat. The experimental schedule is as **Figure 1**.

### 2.5 Assessment of CIA-associated symptoms

During the experiment, the weight of the rats was measured weekly. Since the arthritic symptoms developed at paw and joint, the paws thickness was measured using calipers and the severity of symptoms was assessed by an arthritis clinical score following the method described previously (13). The quantitative clinical score is as follows: 0, no signs of erythema and swelling; 1, erythema and mild swelling limited to the tarsal bone or ankle joint; 2, erythema and mild swelling extending from the ankle joint to the tarsal bone; 3, Erythema and moderate swelling extending from the ankle joint to the metatarsal joint; 4, erythema and severe swelling of the ankles, feet, and fingers, or stiffness of the limbs.

### 2.6 Histopathology analysis

Clinical condition and symptoms of each rat were evaluated by histological analysis with haematoxylin and eosin (H&E) and safranin O-fast green staining. At the end of the experiment on the 63rd day, the rats were sacrificed and the knee joints were taken. The joints were fixed in 10% formalin for 48 h, decalcified in 10% EDTA for 30 days, and embedded in paraffin. Tissue sections (5 µm thick) in the sagittal direction along the long axis of the knee were stained using H&E and safranin O-fast green staining.

### 2.7 Immunofluorescent staining

Formalin-fixed paraffin-embedded samples (5 µm thick) were deparaffinized and retrieved with bone tissue antigen retrieval solution was in a wet box at 37°C for 2 h. After sealed at room temperature for 1 h, samples were then incubated with the primary antibody (anti TNF-α and anti MMP-13, respectively) (Abcam, Cambridge, UK) at 4°C overnight, followed by reaction with goat anti-rabbit secondary antibody (FITC) (Abcam, Cambridge, UK) at room temperature for 1 h in the dark. After washing, 4`,6-diamidino-2-phenylindole (DAPI) was applied for re-dyeing and incubated at room temperature for 5 min in the dark, and then the sample was sealed and observed under fluorescence microscope.

### 2.8 Measurement of collagen-specific IgG and inflammatory factors by ELISA in serum and joint fluid

Total II collagen-specific IgG (CII-IgG), and its subclasses CII-IgG1, CII-IgG2a and CII-IgG2b were detected by ELISA kits according to the manufacturer`s instructions. Inflammatory factors interleukin-1β (IL-1β), interleukin-17A (IL-17A) and tumor necrosis factor-α (TNF-α) in serum and synovial fluid were detected using an ELISA kit according to the manufacturer’s instructions (Hangzhou LianKe Biological Technology Co., Ltd, Hangzhou, China). OD values were measured by ELIASA.

### 2.9 Quantitative real-time PCR

The transcription levels of ZO-1, Occludin-1, Claudin-1 and MUC-2 in rat ileum were measured via qPCR. Total RNA from rat ileum tissue was extracted using TRIzol reagent (Invitrogen Life Technologies, Carlsbad, CA). Then, total RNA was analyzed as described previously (14). The primers used are shown in **Table 2**. Analysis of each sample was repeated three times. β-actin RNA was used as the endogenous control.

**Table 2:** Primers used in quantitative real-time PCR.

Genes	Primer Sequence (5`-3`)
β-actin	Forward primer: TCAGGTCATCACTATCGGCAAT
	Reverse primer: AAAGAAAGGGTGTAAAACGCA
ZO-1	Forward primer: TTTTTGACAGGGGGAGTGG
	Reverse primer: TGCTGCAGAGGTCAAAGTTCAAG
Occludin-1	Forward primer: GTCTTGGGAGCCTTGACATCTT
	Reverse primer: GCATTGTCGAACGTGCATC
Claudin-1	Forward primer: GGGACAACATCGTGACTGCT
	Reverse primer: CCACTAATGTCGCCAGACCTG
MUC-2	Forward primer: AGATCCCGAAACCATGTC
	Reverse primer: GTTCCACATGAGGGAGAGG

### 2.10 Analysis of the microbial community

Fecal samples were collected at the end of the experiment and stored at –80°C. Total fecal DNA was extracted using the TIANamp Bacteria DNA Kit (TIANGEN Biotech, Beijing, China) according to the manufacturer’s instructions. The V3-V4 region of 16S rDNA was amplified using primer set 341F/806R and sequenced using a MiSeq sequencer (Illumina, San Diego, CA, USA). The microbial communities were further predicted by PICRUSt.

### 2.11 Fecal metabolomics

#### 2.11.1 Metabolites extraction

The fecal samples (25 mg ± 1 mg) were taken, mixed with beads and 500 μL of extraction solution (MeOH: CAN: H_2_O, 2:2:1 (v/v)). The extraction solution contains deuterated internal standards. The mixed solution was vortexed for 30 s. These soil samples (100 mg ± 1 mg) were taken, mixed with beads and 500 μL of extraction solution (MeOH: CAN: H_2_O, 2:2:1 (v/v)). The extraction solution contains deuterated internal standards. The mixed solution was vortexed for 30 s.

Then the mixed samples were homogenized (35 Hz, 4 min) and sonicated for 5 min in 4°C water bath, the step repeat for three times. The samples were incubated for 1 h at -40°C to precipitate proteins. Then the samples ware centrifuged at 12000 rpm (RCF=13800(×g), R= 8.6cm) for 15 min at 4°C. The supernatant was transferred to a fresh glass vial for analysis. The quality control (QC) sample was prepared by mixing an equal aliquot of the supernatant of samples.

#### 2.11.2 LC-MS analysis

For polar metabolites, LC-MS/MS analyses were performed using an UHPLC system (Vanquish, Thermo Fisher Scientific) with a Waters ACQUITY UPLC BEH Amide (2.1 mm × 50 mm, 1.7 μm) coupled to Orbitrap Exploris 120 mass spectrometer (Orbitrap MS, Thermo). The mobile phase consisted of 25 mmol/L ammonium acetate and 25 ammonia hydroxide in water(pH = 9.75)(A) and acetonitrile (B). The auto-sampler temperature was 4°C, and the injection volume was 2 μL. The Orbitrap Exploris 120 mass spectrometer was used for its ability to acquire MS/MS spectra on information-dependent acquisition (IDA) mode in the control of the acquisition software (Xcalibur, Thermo). In this mode, the acquisition software continuously evaluates the full scan MS spectrum. The ESI source conditions were set as following: sheath gas flow rate as 50 Arb, Aux gas flow rate as 15 Arb, capillary temperature 320°C, full MS resolution as 60000, MS/MS resolution as 15000, collision energy: SNCE 20/30/40, spray voltage as 3.8 kV (positive) or -3.4 kV (negative), respectively.

#### 2.11.3 Data preprocessing and annotation

The raw data were converted to the mzXML format using ProteoWizard and processed with an in-house program. which was developed using R and based on XCMS, for peak detection, extraction, alignment, and integration. The R package and the BiotreeDB(V3.0) were applied in metabolite identification (15).

### 2.12 Statistical analysis

All statistical analyses were performed using SPSS 19.0 statistical software (SPSS Inc., Chicago, IL, USA). The results were expressed as mean ± standard deviation. Statistical differences among the groups were assessed by one-way ANOVA, and multiple comparisons were performed using Tukey HSD test. Values of *p* < 0.05 were considered statistically significant.

## 3 Results

### 3.1 *Bifidobacterium* significantly alleviates experimental arthritis

**Figure 2:**
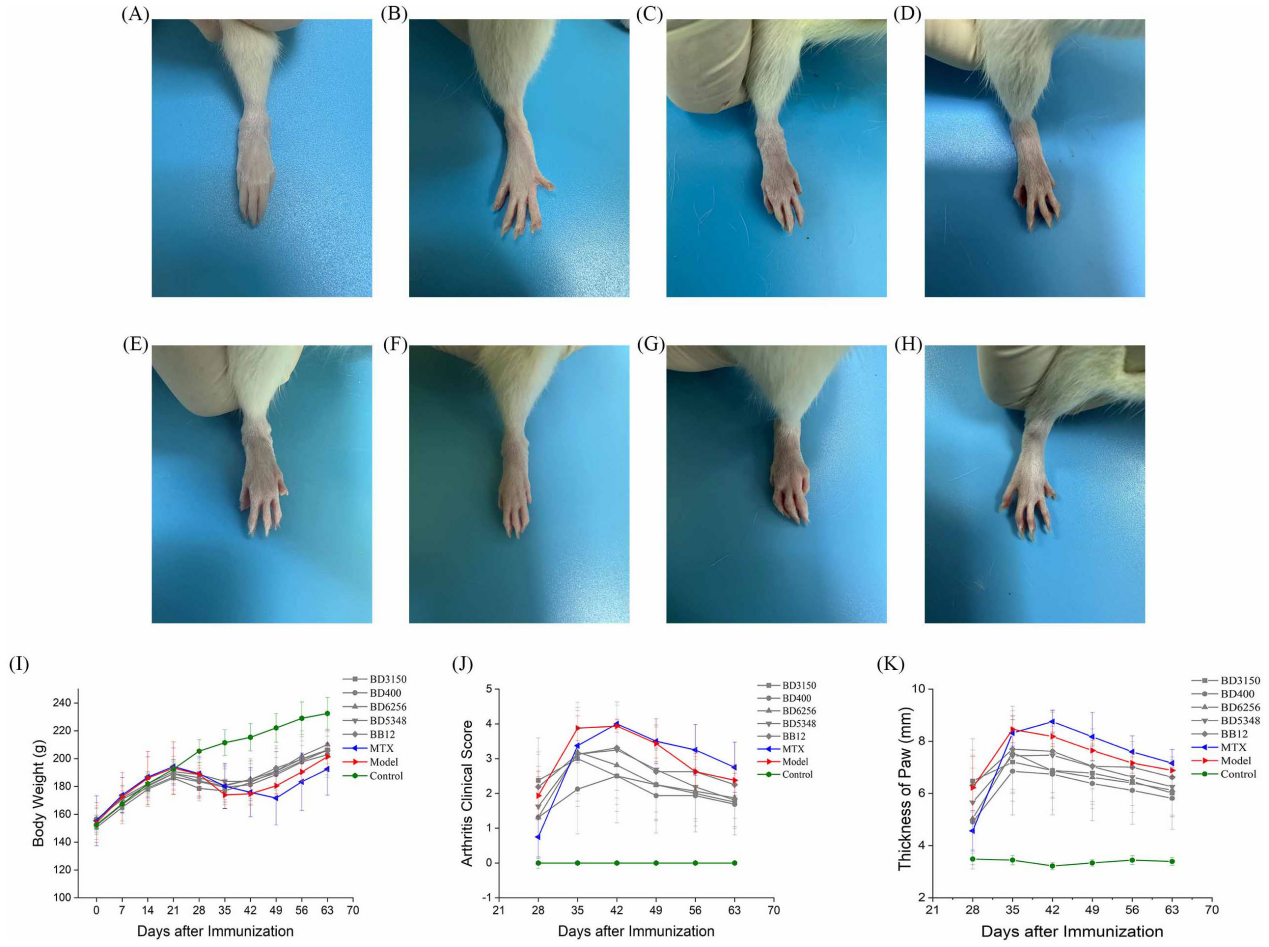
Effect of oral *Bifidobacterium* on the symptoms of paw arthritis in CIA rats. **(A)** *B. longum* BD3150, **(B)** *B. animalis* BD400, **(C)** *B. longum* BD6256, **(D)** *B. bifidum* BD5348, **(E)** *B. breve* BB12, **(F)** MTX, **(G)** CIA model, **(H)** Control, **(I)** Body weight, **(J)** Arthritis clinical score, **(K)** Thickness of paw. (n=8).

After a seven-day acclimatization period, Wistar rats were administered either a *Bifidobacterium* solution or sterile saline orally on day 0. The CIA model group, MTX group, and *Bifidobacterium* solution group were induced as CIA models with primary immunization on day 14 and booster immunization on day 21. The *Bifidobacterium* solution group received oral gavage of *Bifidobacterium* solution (2 × 10^8^ CFU) from day 0 to day 63. The CIA model and control groups were orally gavaged with sterile saline from day 0 to day 63. The MTX group received sterile saline orally from day 0 to day 63 and was treated with MTX twice a week from day 14 to day 63 (**Figure 1**). Paw photos of each group of rats on day 63 are shown in **Figure 2**. A comparison between **Figures 2G, H** reveals significant redness and swelling in the feet and fingers of the CIA rats. The *Bifidobacterium* intervention demonstrated a reduction in this redness and swelling (**Figures 2A–E**). In MTX group, before day 35, the clinical score and paw thickness of the rats were lower than those of the CIA model group. After 35 days, the MTX group and the CIA model group had similar results. This result in the MTX group is related to the properties of the drug MTX. Treatment of RA with MTX is often done at an early stage, which is more beneficial for controlling the progression of the disease early on. In addition, in the treatment of MTX, the response rate is 64%-87%. Combined with the above two reasons, MTX showed poor performance in reducing clinical score and paw swelling (16).

The study recorded the body weight, clinical scores, and paw thickness of rats to evaluate the severity of arthritis. Prominent symptoms of CIA rats, such as swelling and reddening, appeared on day 21. As depicted in **Figure 2I**, the weight of CIA rats began to decrease, but by day 35, it had returned to an upward trend as RA stabilized. In contrast, the control group’s rat weight steadily increased. The MTX group’s rat weight hit its lowest point on day 49. Between days 42 and 63, the MTX group’s rat weight was consistently lower than that of the CIA model group, likely due to MTX’s hepatotoxic nature causing gastrointestinal discomfort like vomiting and diarrhea. This resulted in lower body weight compared to the model group. Similarly, the weight of rats in the *Bifidobacterium* groups (BD3150, BD400, BD6256, BD5348, BB12) exhibited a pattern of initial increase, subsequent decrease, and final rise. There were minimal differences in rat weight among the *Bifidobacterium* groups.

Arthritis onset was observed in rats one week after booster immunization, with arthritis clinical scores and paw thickness recorded (**Figures 2J, K**). Paw swelling in CIA rats continued to increase from day 28 to day 42, peaking on day 42 before entering a stable phase with gradual reduction in swelling. While MTX intervention did not show immediate effects in early arthritis stages, it did reduce paw swelling in later stages. Disease activity persisted after MTX treatment, a common scenario in RA management (17). *Bifidobacterium* administration reduced paw swelling in CIA rats, with the most significant difference seen in the *B. animalis* BD400 group.

### 3.2 Histopathological analysis of CIA rats` joint improved by *Bifidobacterium*

**Figure 3:**
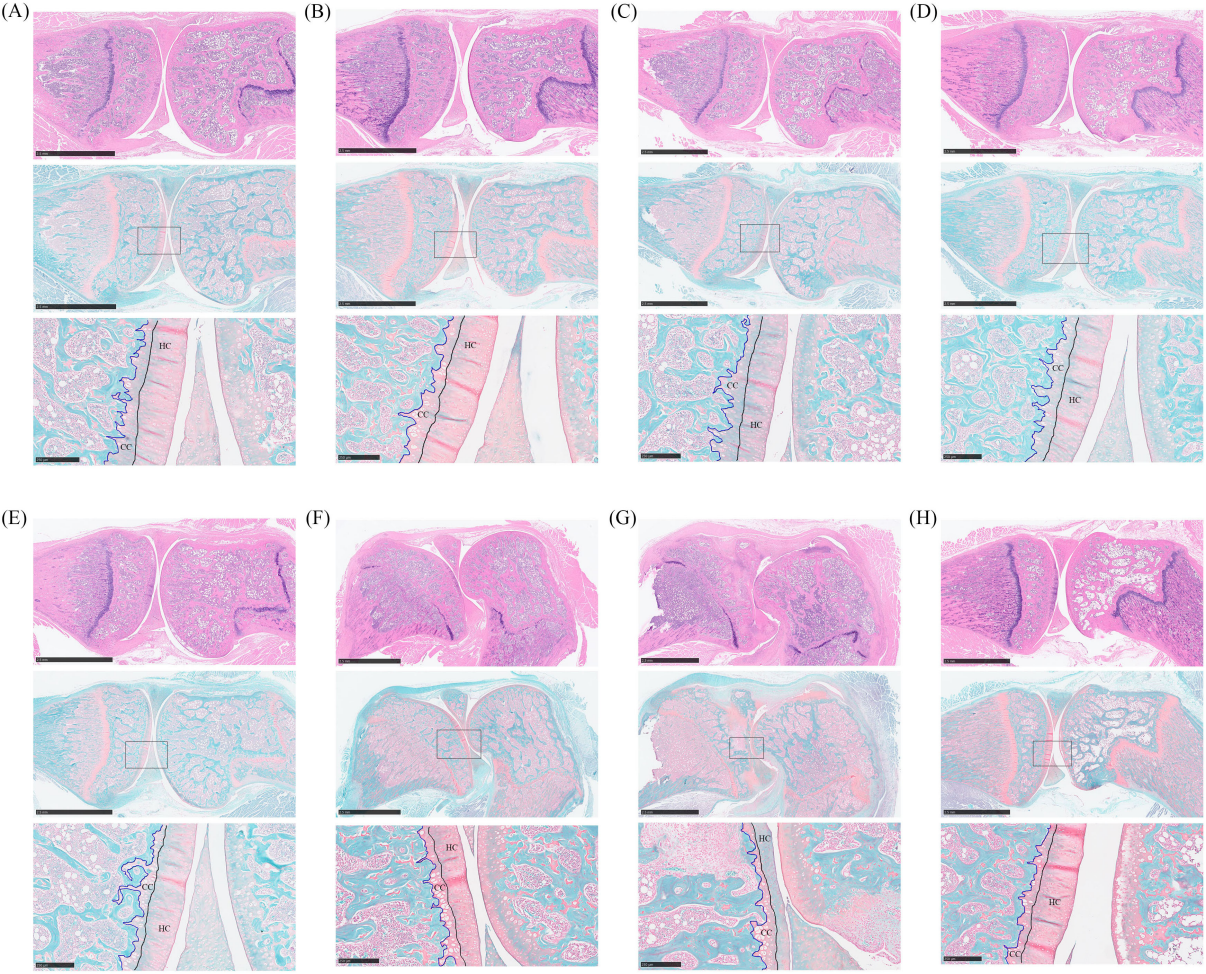
Histopathological analysis of oral treatment with *Bifidobacterium* in CIA rats. From the top down are the HE staining picture, the safranin O-fast green staining picture, and the enlarged picture at the box of the safranin O-fast green staining picture. **(A)** *B. longum* BD3150, **(B)** *B. animalis* BD400, **(C)** *B. longum* BD6256, **(D)** *B. bifidum* BD5348, **(E)** *B. breve* BB12, **(F)** MTX, **(G)** CIA model, **(H)** Control.

The knee joints of rats were collected for histopathological analysis. Bone erosion in the knee joints was observed through HE staining, while cartilage damage was assessed using safranin O-fast green staining. In **Figure 3H**, joint staining in normal rats appeared uniform, with round chondrocytes evenly arranged and abundant extracellular collagen fibers. Conversely, the knee bones of CIA rats showed severe damage, with less stained cartilage matrix indicating significant collagen loss, along with damage to the synovial membrane and cartilage (**Figure 3G**). Rats in the *Bifidobacterium* and MTX groups exhibited uniformly colored joints and cartilage, suggesting restoration of inflammatory damage and collagen loss in the joints (**Figures 3A–F**).

Safranin O-fast green staining can reveal cartilage damage by coloring normal cartilage layers, hyaline cartilage (HC) and calcified cartilage (CC), in red, while coloring cancellous bone layers in green. HC contains active chondrocytes responsible for repairing the cartilage matrix, whereas CC represents mineralized HC. Thicker CC layers can impede the flow of liquid and small organic molecules into HC, hindering chondrocyte growth (18). In the study, normal rats exhibited thick and intact HC layers with a clearly visible tidal line, along with moderately thick CC layers (**Figure 3H**). Conversely, rats in the CIA model group showed reduced HC thickness, intensified cartilage matrix degradation, and thickened mineralized CC layers (**Figure 3G**). Treatment with *Bifidobacterium* increased HC thickness and decreased CC thickness in CIA rats, indicating a reduction in inflammation levels that promoted chondrocyte biological activity and alleviated RA development (**Figures 3A–E**). Specifically, the *Bifidobacterium* group showed knee bone and cartilage conditions similar to those of the normal group, with both *B. longum* BD3150 and *B. animalis* BD400 groups exhibiting comparable results.

### 3.3 Expression of inflammatory markers in rat joint tissues

**Figure 4:**
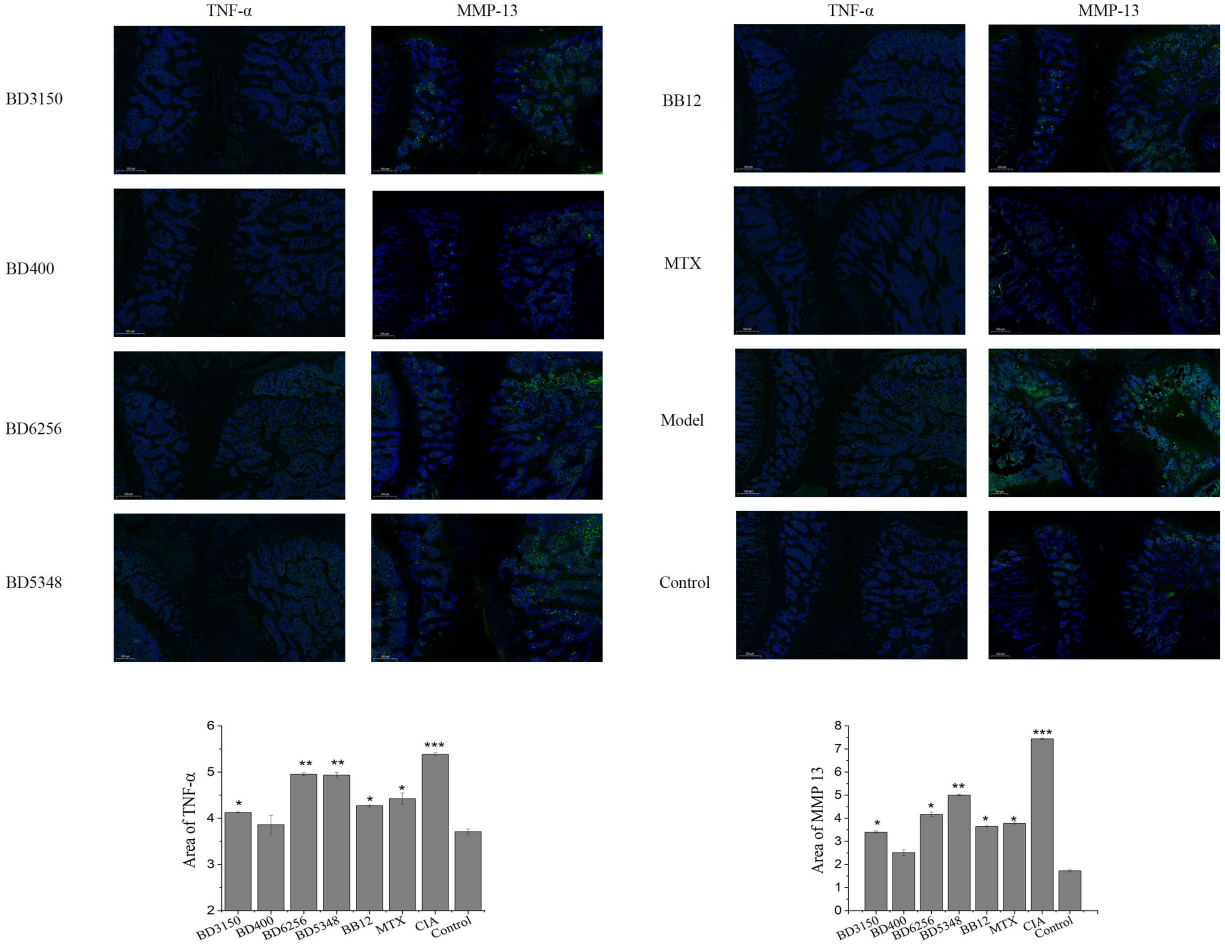
Expression of TNF-α and MMP13 in rat joint tissues. (***p < 0.01, **p < 0.01, *p < 0.05 against the control group.) (n=8).

Immunofluorescence staining was conducted on histopathologic sections of rat knee joints to examine TNF-α and MMP-13 distribution (**Figure 4**). In the control group, articular cartilage appeared normal with no positive fluorescence. However, in the CIA model group, increased TNF-α and MMP-13 proteins were observed. Treatment with *Bifidobacterium* led to reduced protein expression, particularly in the *B. animalis* BD400 group. Quantitative analysis using Image J software showed significantly higher fluorescence in the CIA model group compared to controls (*p* < 0.05). *Bifidobacterium* intervention decreased fluorescence significantly (*p* < 0.05), with no difference between the *B. animalis* BD400 group and control group (*p* > 0.05). The details of the comparison of significant differences in the **Supplementary Table S3.**

### 3.4 Effect of *Bifidobacterium* on specific antibody of CIA rats

**Figure 5:**
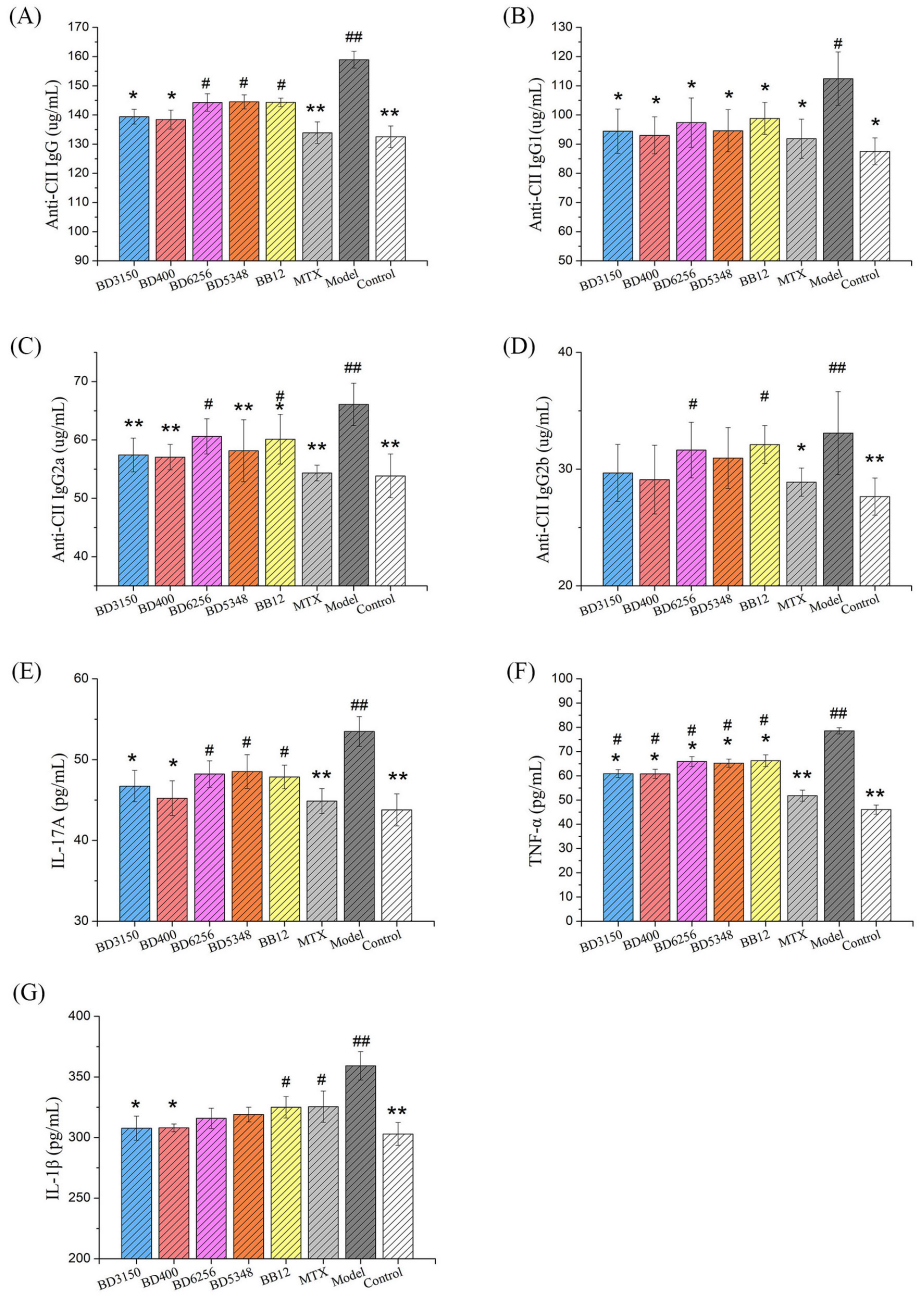
Effects of *Bifidobacterium* on inflammatory responses in rats. The levels of anti-CII IgG **(A)**, anti-CII IgG1 **(B)**, anti-CII IgG2a **(C)**, anti-CII IgG2b **(D)** and IL-17A **(E)** in serum were examined. The levels of TNF-α **(F)** and IL-1β **(G)** in the synovial fluid were examined. (^##^ *p* < 0.01, ^#^ *p* < 0.05 against the control group; **p < 0.01, *p < 0.05 against the CIA group as determined by a two-way ANOVA with Tukey`s multiple comparisons.) (n=8).

Anti-CII IgG is a specific antibody that targets type II collagen and plays a crucial role in the development of arthritis in CIA rats. It is known to induce or worsen arthritis and can be used to evaluate the severity of the disease. Lowering the production of these autoantibodies can effectively halt the progression of arthritis (19). In this study, the impact of *Bifidobacterium* on the humoral immunity of CIA rats was assessed by measuring the levels of CII-specific antibodies. The study measured the levels of total anti-CII IgG as well as its subtypes (anti-CII IgG1, IgG2a, IgG2b) in the serum. Results showed that the levels of these antibodies were significantly higher in the CIA model group compared to the control group (**Figures 5A–D**). Treatment with MTX reduced these antibody levels to a level comparable to that of normal rats. Oral administration of *Bifidobacterium* in CIA rats also led to a notable decrease in these antibody levels. Specifically, the *B. longum* BD3150 and *B. animalis* BD400 groups showed significantly lower levels of total anti-CII IgG and its subtypes compared to the CIA model group. These findings suggest that *B. longum* BD3150 and *B. animalis* BD400 may help alleviate inflammatory damage induced by autoantibodies by reducing the levels of anti-CII IgG, anti-CII IgG1, and anti-CII IgG2a. The details of the comparison of significant differences in the **Supplementary Table S3.**

### 3.5 Effect of *Bifidobacterium* on immune responses of CIA rats

Cytokine levels in serum serve as indicators of systemic inflammatory immune response, reflecting the overall inflammatory status of the body. Notably, IL-1 and TNF-α are key inflammatory mediators in RA, with IL-17 playing a significant role in promoting the secretion of IL-1 and TNF-α by inflammatory cells. In the study (**Figure 5E**), levels of IL-17A in the serum of the CIA model group were markedly higher compared to other groups (*p* < 0.01). Treatment with MTX led to a significant reduction in IL-17A levels in the serum of CIA rats (*p* < 0.01). Furthermore, administration of *B. longum* BD3150 and *B. animalis* BD400 to CIA rats resulted in a significant decrease in IL-17A levels in serum (*p* < 0.05) (**Figures 5F, G**). Additionally, analysis of synovial fluid from rats revealed elevated levels of TNF-α and IL-1β in the CIA model group compared to control groups (*p* < 0.01). Treatment with *B. longum* BD3150 and *B. animalis* BD400 significantly lowered TNF-α and IL-1β levels in the synovial fluid of CIA rats (*p* < 0.05). These findings suggest that *B. longum* BD3150 and *B. animalis* BD400 effectively reduce inflammation in both the blood and joint fluid of CIA rats. The details of the comparison of significant differences in the **Supplementary Table S3.**

### 3.6 Effects of *Bifidobacterium* on intestinal barrier related protein genes in CIA rats

**Figure 6:**
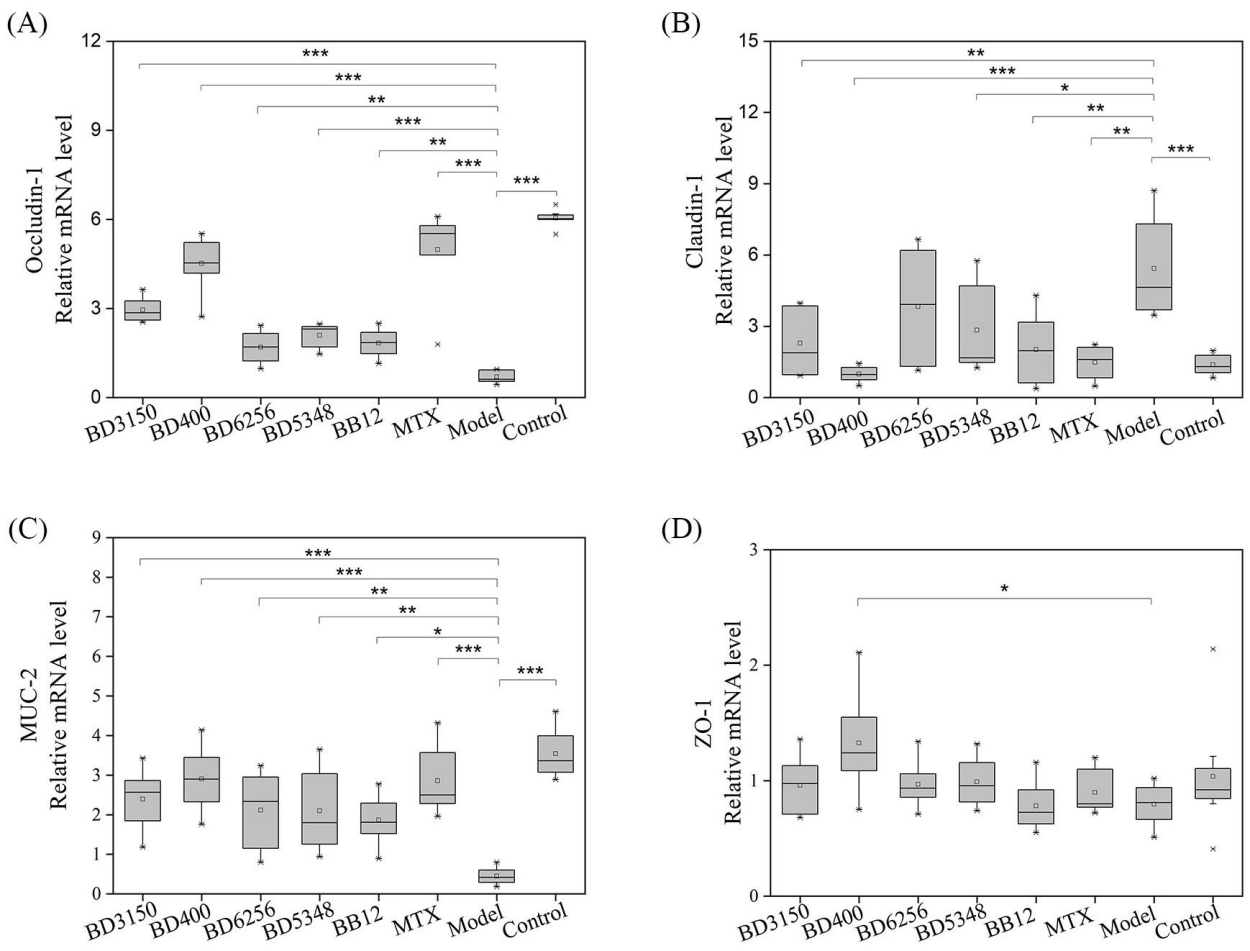
Intestinal barrier protein gene expression in rats. **(A)** occludin-1, **(B)** claudin-1, **(C)** MUC-2, **(D)** ZO-1. (*p < 0.05, **p < 0.01, ***p < 0.001 against the CIA model group as determined by a two-way ANOVA with Tukey`s multiple comparisons.) (n=8).

Intestinal barrier proteins, such as claudin-1, occludin-1, mucin-2 (MUC-2), and zonula occluden-1 (ZO-1), play a crucial role in maintaining normal intestinal barrier function. Gene expression analysis in a CIA model group revealed a significant decrease in occludin-1 and MUC-2 levels, an increase in claudin-1 expression, and no notable change in ZO-1 levels compared to the control group (**Figure 6**). Treatment with *Bifidobacterium* led to a significant increase in occludin-1 and MUC-2 expression, while claudin-1 levels decreased. In the *B. animalis* BD400 group, there was a significant increase in occludin-1, MUC-2, and ZO-1 expression, with a decrease in claudin-1 levels. Occludin-1 regulates intercellular ion transport, while MUC-2 is a mucin produced by goblet cells in the intestinal wall. The modulation of occludin-1 and MUC-2 by *B. animalis* BD400 can enhance intestinal selective permeability, reduce the influx of harmful substances into the internal environment, and maintain internal homeostasis. The details of the comparison of significant differences in the **Supplementary Table S3.**

### 3.7 Effects of *Bifidobacterium* on the composition and diversity of gut microbiota

**Figure 7:**
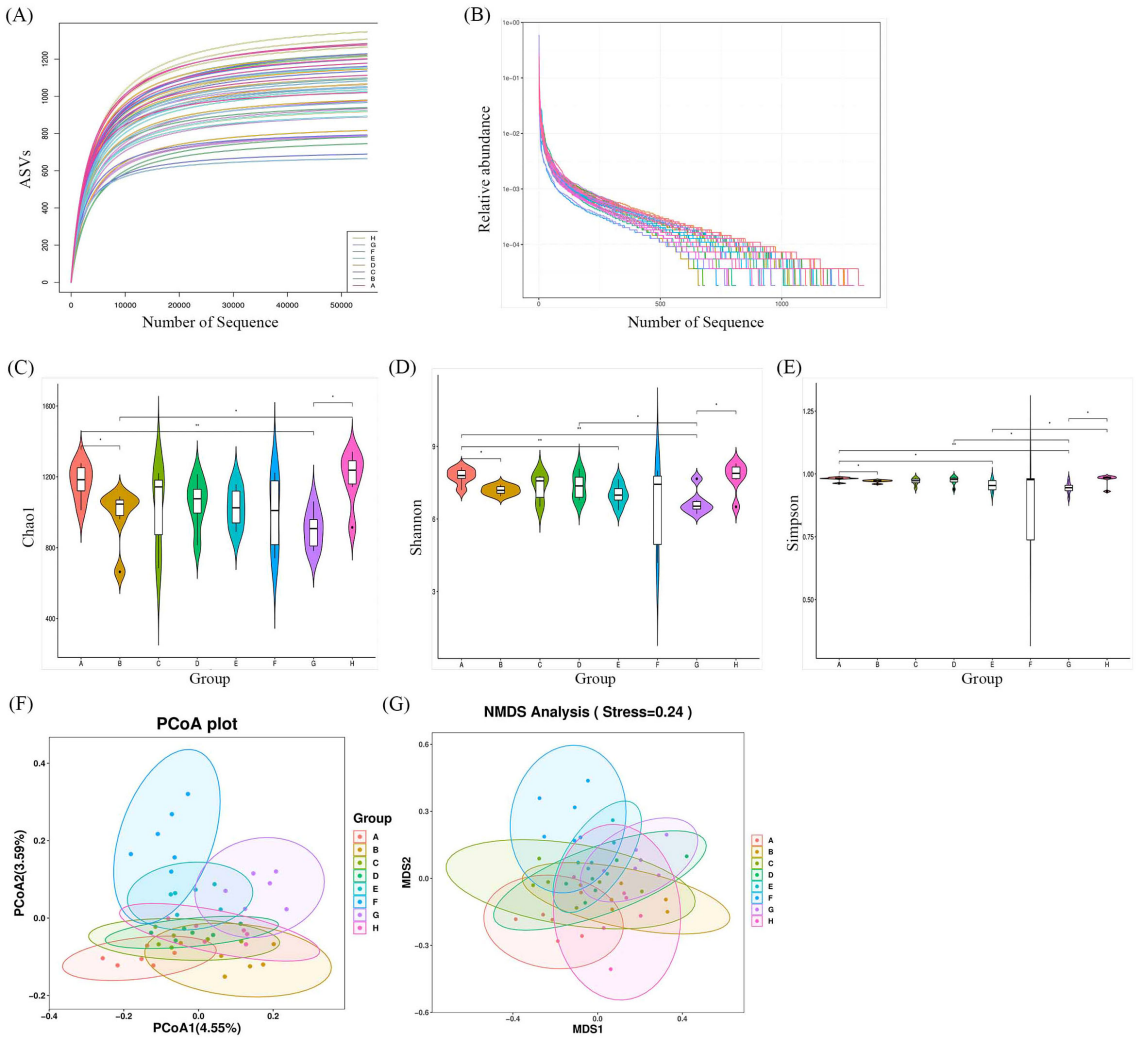
Effects of *Bifidobacterium* on the relative abundance and diversity of gut microbiota. **(A)** Specaccum curve, **(B)** Rank-abundance curve, **(C)** Chao1, **(D)** Shannon, **(E)** Simpson, **(F)** PCoA, **(G)** NMDS. Group: (A): *B. longum* BD3150, (B): *B. animalis* BD400, (C): *B. longum* BD6256, (D): *B. bifidum* BD5348, (E): *B. breve* BB12, (F): MTX, (G): CIA Model, (H): Control. (*p < 0.05, **p < 0.01, determined by a two-way ANOVA.) (n=6).

Faecal samples were collected to evaluate the impact of *Bifidobacterium* on the gut microbiota of CIA rats through 16S rDNA V3-V4 sequencing. A total of 3,276,658 valid sequences were obtained after double-end splicing, quality control, and chimera filtering. Through amplicon sequence variants (ASVs) cluster analysis and species annotation, 50,065 ASVs were identified, with an average of 1,043 ASVs per sample. The species accumulation curve in **Figure 7A** shows a plateau as sample size increases, indicating sufficient sampling. Additionally, the dilution curve in **Figure 7B** flattens with the increase in sample sequences, suggesting an appropriate amount of sequencing data. Significant differences in Chao1 index, Shannon index, and Simpson index were observed between the CIA model group and the control group (*p* < 0.05) in **Figures 7C–E**. Following the intervention of certain *Bifidobacterium* strains, there was a notable increase in species richness within the gut microbiota of CIA rats (*p* < 0.05). Specifically, the Chao1 index, Shannon index, and Simpson index significantly increased in the *B. longum* BD3150 group (*p* < 0.01), while the Shannon index and Simpson index notably increased in the *B. bifidum* BD5348 group (*p* < 0.05). These findings indicate substantial disparities in species diversity within the gut microbiota of CIA rats compared to normal rats, and highlight the potential of *B. longum* BD3150 and *B. bifidum* BD5348 interventions in mitigating the reduction in intestinal species diversity induced by arthritis.

Principal coordinate analysis (PCoA) and nonmetric multidimensional scaling (NMDS) were utilized to compare the distribution profiles of fecal microorganisms in eight groups. **Figures 7F, G** demonstrates a significant difference in PCoA and NMDS between the CIA model group (purple circle) and the control group (pink circle). Upon administration of *Bifidobacterium*, there were significant alterations in the distribution profiles, particularly in the *B. longum* BD3150 group and the *B. animalis* BD400 group in PCoA. Adonis analysis revealed a significant difference in the species composition of gut microbiota among the eight groups (*p* = 0.001) (**Table 3**).

**Table 3:** Adonis multivariate analysis based on Bray-Curtis distance.

	Df	Sum of sqs	R^2^	F	*p*
Group	7	2.57	0.28	2.25	0.00
Residual	40	6.52	0.72		
Total	47	9.09	1		

Df, Degree of freedom; Sum of sqs, sum of squares of deviations; R^2^, R^2^ represents the interpretation of sample differences by different groups, that is, the ratio of group variance to total variance; F, F test value; *p*, *p* value, *p* < 0.05 indicates a high degree of reliability. n=6.

**Figure 8:**
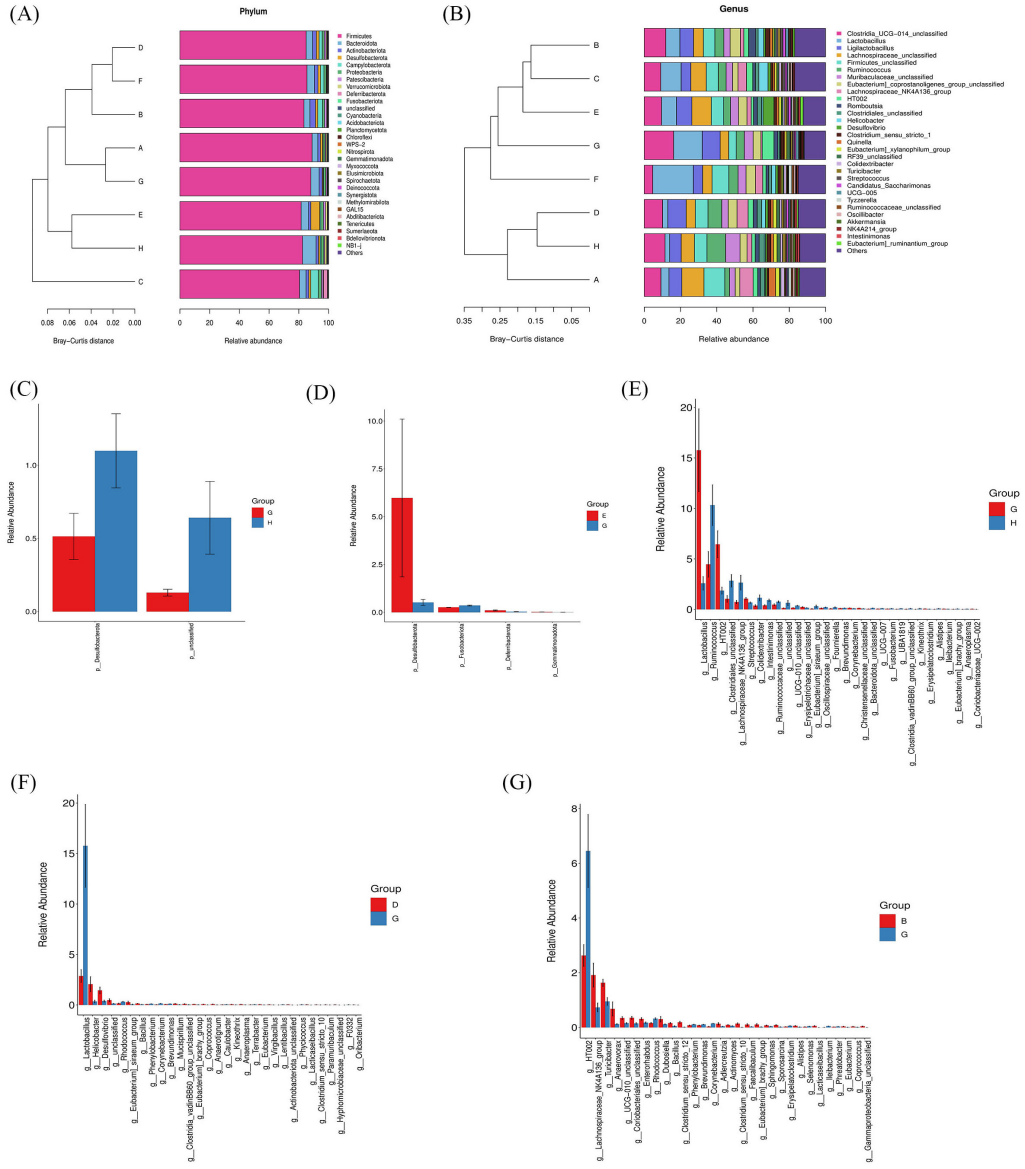
Effects of *Bifidobacterium* on the species composition of gut microbiota. Species abundance at the phylum level **(A)**, genus level **(B)**, and a relative abundance difference between group G and group H at the phylum level **(C)**, between group E and group G at the phylum level **(D)**, between group G and group H at the genus level **(E)**, between group D and group G at the genus level **(F)**, between group B and group G at the genus level **(G)**. Group: (A): *B. longum* BD3150, (B): *B. animalis* BD400, (C): *B. longum* BD6256, (D): *B. bifidum* BD5348, (E): *B. breve* BB12, (F): MTX, (G): CIA Model, (H): Control. (n=6).

Based on ASV annotation results and the ASV abundance table for each sample, we generated a species abundance table at various taxonomic levels including kingdom, phylum, class, order, family, genus, and species. Utilizing this information along with species annotation data, we identified the top 30 species for constructing cluster stacked bar charts at the phylum and genus levels. In **Figure 8A**, *Firmicutes* and *Bacteroidota* emerge as the predominant phyla in rat gut bacteria, with a higher proportion observed in the CIA model group compared to the control group. Following intervention with specific strains of *Bifidobacterium*, the ratio of *Firmicutes* and *Bacteroidota* exhibited alterations. Notably, clustering based on Bray-Curtis distance revealed that the *B. breve* BB12 group closely resembled the control group in terms of *Firmicutes* and *Bacteroidota* percentages. Moving to the genus level (**Figure 8B**), the top 5 species included *Clostridia_UCG-014*_unclassified, *Lactobacillus*, *Ligilactobacillus*, *Lachnospiraceae*_unclassified, and *Firmicutes*_unclassified. The proportion of these species differed significantly between the CIA model group (50.72%) and the control group (34.57%) (*p* < 0.05). Upon *Bifidobacterium* administration, a decrease in the percentages of these top 5 species was observed, particularly in the *B. bifidum* BD5348 group.

To further investigate the species contributing to the differences between groups, a differential abundance analysis was conducted. In **Figure 8C**, *Desulfobacterota* at the phylum level exhibited the most significant difference between the CIA model group and the control group. Following the administration of *B. breve* BB12, there was an increase in the relative abundance of *Desulfobacterota* (**Figure 8D**). At the genus level, *Lactobacillus* showed the most significant difference between the CIA model group and the control group (**Figure 8E**). Additionally, *Ruminococcus* and HT002 were also found to differ between the CIA model group and the control group. Notably, there was no observed increase in *Ruminococcus* with the administration of *Bifidobacterium*, while *B. animalis* BD400 reduced the relative abundance of HT002.

**Figure 9:**
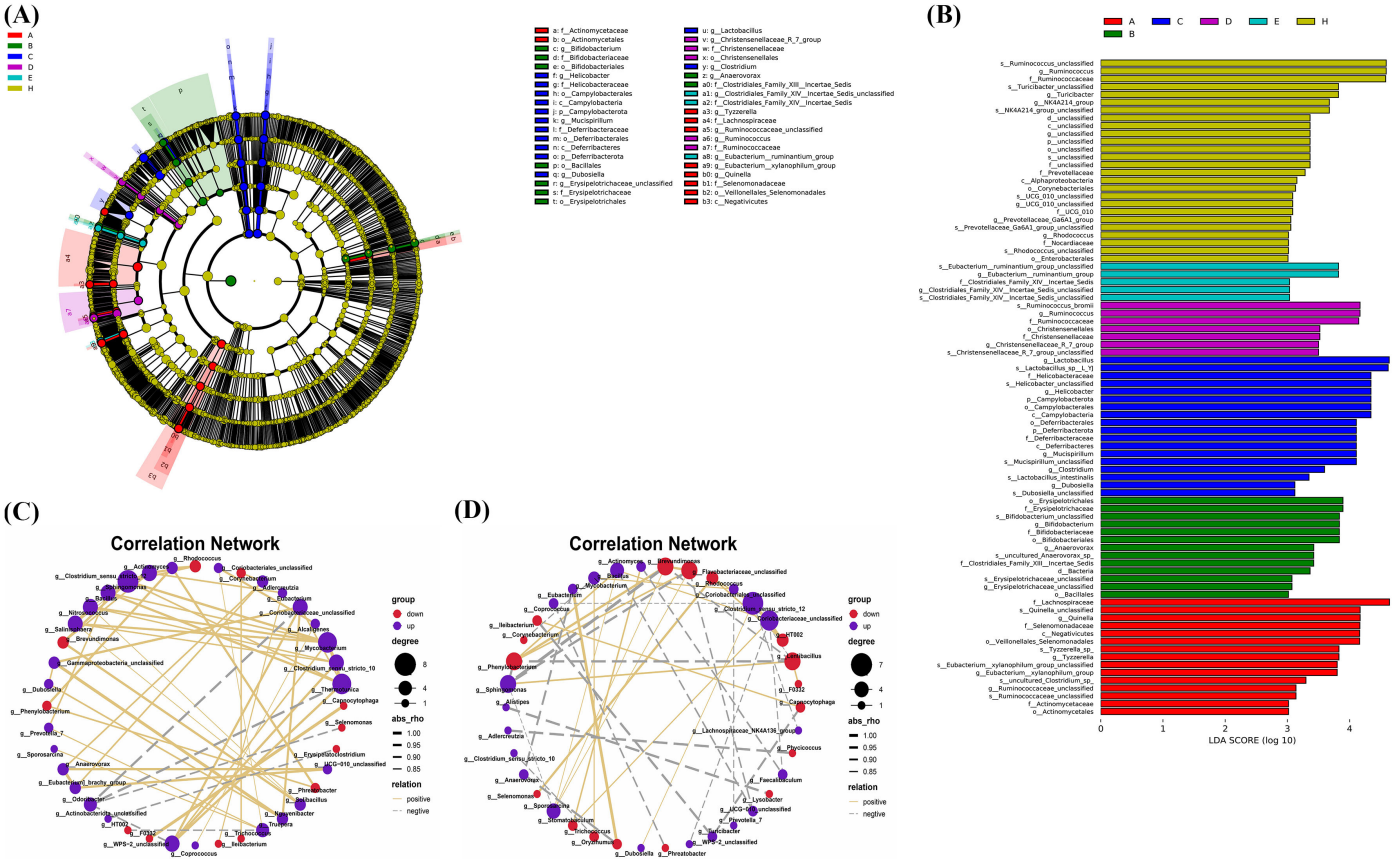
LEfSe analysis and correlation analysis. The cladogram **(A)** and the bar diagram **(B)** of LEfSe analysis. The correlation analysis between significantly up-regulated and significantly down-regulated differential species in the *B. animalis BD400* group **(C)** and the CIA model group **(D)**. (n=6).

The LEfSe tool was utilized to identify biomarkers between groups. In **Figures 9A, B**, 26 types of bacteria showed significant differences across various classification levels including phylum, class, order, family, and genus in the control group. The top 5 bacteria with notable differences were *Ruminococcus*_unclassified, *Ruminococcus*, *Ruminococcaceae*, *Turicibacter*_unclassified, and *Turicibacter*. In the *B. bifidum* BD5348 group, 7 types of bacteria exhibited significant differences, namely *Ruminococcus*_bromii, *Ruminococcus*, *Ruminococcaceae*, *Christensenellales*, *Christensenellaceae*, *Christensenellaceae*_R_7_group, and *Christensenellaceae*_R_7_group_unclassified. These results indicate that both the *B. bifidum* BD5348 group and the control group share biomarkers such as *Ruminococcus* and *Ruminococcaceae*, suggesting that the intervention of *B. bifidum* BD5348 alters the gut microbiota of CIA rats to resemble that of normal rats.

**Figures 9C, D** were obtained by Spearman correlation analysis. Comparing the two correlation networks, it was found that most of the significant different bacteria genera in the CIA model group were negatively correlated. After *B. animalis* BD400 intervention, the correlation was mainly positive. Under the administration of *B. animalis* BD400, *Mycobacterium* was positively correlated with *Sphingomonas*, *Bacillus*, *Nitrosococcus* and *Salinisphaera*. But in the CIA model group, *Mycobacterium* was only positively correlated with *Oryzihumus*. In addition, WPS-2_unclassified was positively correlated with *Coriobacteriaceae*_unclassified, *Thermotunica*, *Clostridium_sensu_stricto*_10 and *Mycobacterium* in the *B. animalis* BD400 group. WPS-2_unclassified in the CIA model group was only negatively correlated with *Coriobacteriaceae*_unclassified.

In addition, in the *B. animalis* BD400 group, genus HT002 was negatively correlated with genus *Truepera* (**Figure 9C**). As mentioned above, the relative abundance of HT002 decreases in the *B. animalis* BD400 group, which means that the relative abundance of *Truepera* increases. *Truperia* comes from *Deinococcota* phylum, *Deinococci* class, *Deinococcales* order, *Trueperaceae* family. *Trueperaceae* is a relatively newly defined family of bacteria. In 2005, Albuquerque et al. discovered and described a new genus of *Trueperaceae* family, confirming its taxonomic status and laying the foundation for the establishment of Trueperaceae family (20, 21). Little is known about the physiological role of *Truperia*. In the CIA model group, genus HT002 was positively correlated with genus *Rhodococcus*. As mentioned above, the relative abundance of HT002 increases in the CIA model group, which means that the relative abundance of *Rhodococcus* increases. *Rhodococcus* comes from *Actinobacteriota* phylum, *Actinobacteria* class, *Corynebacteriales* order, *Nocardiaceae* family. Some species of *Rhodococcus* are human and animal pathogens, such as *Rhodococcus equi* is a pathogen of horses, pigs, cattle and other animals (22). In summary, we hypothesized that HT002 can disrupt the homeostasis of gut microbiota by promoting the growth of harmful bacteria in gut microbiota. *B. animalis* BD400 can reduce the relative abundance of HT002 and restore gut microbiota homeostasis.

### 3.8 Untargeted metabolomics of gut microbiota

A total of 11632 peaks in positive ion mode and 9769 peaks in negative ion mode were retained after quality control. Differential metabolites between the control and treatment groups were identified based on the variable importance in the projection (VIP) of the OPLS-DA model. Subsequently, a T-test was conducted using SPSS 19.0 statistical software to perform statistical analysis. The criteria for screening differential metabolites included ∣Log_2_ FOLD CHANGE∣>1, *p* < 0.01 and VIP > 1. The ratio of each differential metabolite was calculated and transformed logarithmically with a base of 2.

**Figure 10:**
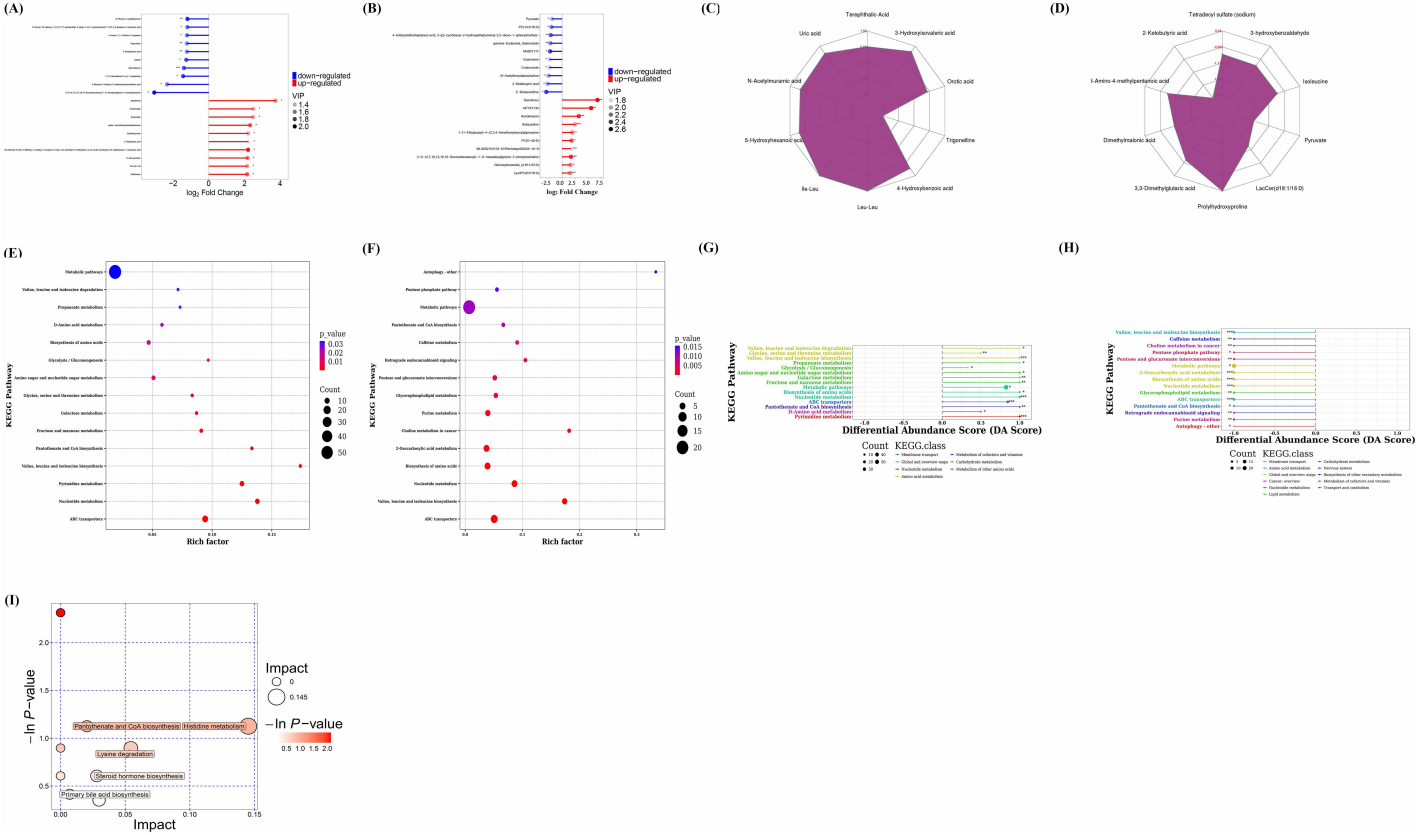
Analysis of differential metabolites and metabolic pathways via non-targeted metabolomics. Matchstick diagram analysis of differential metabolites for CIA model group vs control group **(A)**, for *B. animalis* BD400 group vs control group **(B)**. Radar chart analysis of differential metabolites for CIA model group vs control group **(C)**, for *B. animalis* BD400 group vs control group **(D)**. Bubble plot of KEGG Enrichment for CIA model group vs control group **(E)**, for *B. animalis* BD400 group vs control group **(F)**. Differential Abundance Score for CIA model group vs control group **(G)**, for *B. animalis* BD400 group vs control group **(H)**. Bubble plot of pathway analysis for CIA model group vs *B. animalis* BD400 group **(I)**. Rich factor, it refers to the ratio of the number of differential metabolites annotated in a pathway to the number of all metabolites in that pathway; DA score, it refers to the ratio of difference between the number of up-regulated and down-regulated differential metabolites annotated on a pathway to the number of all metabolites on that pathway. (n=6).

The comparison of differential metabolites between the CIA model group and the control group was presented in **Figures 10A, B**. Each *Bifidobacterium* group was compared individually with the control group. The results of each *Bifidobacterium* group compared with the control group were shown in the **Supplementary Figures S2**-**S5**. Notably, the changes in differential metabolites between the *B. animalis* BD400 group and the control group were found to be opposite to those between the CIA model group and the control group (**Figure 10B**). For instance, purine nucleosides like crotonoside guanosine, 2’-deoxyuridine, and xanthosine were significantly up-regulated in the CIA model group but down-regulated in the *B. animalis* BD400 group. Similarly, the fatty acid metabolite 2-ketobutyric acid showed significant up-regulation in the CIA model group but down-regulation in the *B. animalis* BD400 group. On the other hand, glycerin phospholipids such as 2-O-(4,7,10,13,16,19-docosahexaenoyl)-1-O-hexadecylglycero-3-phosphocholine, momordicinin, and botulin were significantly down-regulated in the CIA model group but up-regulated in the *B. animalis* BD400 group. These contrasting results between the CIA model group and *B. animalis* BD400 group suggest that *B. animalis* BD400 may possess the ability to mitigate the abnormal differential metabolite profiles associated with RA.

A radar chart (**Figures 10C, D**) was utilized to illustrate the variation trend in the content of differential metabolites. The red font represents the difference multiple for each grid line, while the purple shadow consists of the difference multiple lines for each metabolite. Overall, **Figure 10C** predominantly shows positive difference multiples, indicating an increasing trend, whereas **Figure 10D** mostly displays negative difference multiples, suggesting a decreasing trend. **Figure 10C** compares the content of the same differential metabolite in the CIA model group and the control group, revealing higher levels of certain metabolites in the CIA model group such as orotic acid, 3-hydroxyisovaleric acid, terephthalic acid, uric acid, N-acetylmuramic acid, 5-hydroxyhexanoic acid, Ile-Leu, Leu-Leu, and 4-hydroxybenzoic acid compared to the control group. Conversely, trigonelline was lower in the CIA model group than in the control group. In **Figure 10D**, prolylhydroxyproline in the *B. animalis* BD400 group exhibited higher levels compared to the control group, while 3,3-dimethylglutaric acid, dimethylmalonic acid, 3-amino-4-methylpentanic acid, 2-ketobutyric acid, tetradecyl sulfate, 3-hydroxybenzaldehyde, isoleucine, pyruvate, and LacCer(d18:1/16:0) in the *B. animalis* BD400 group were lower than in the control group. The contrasting trends in difference multiples between the CIA model group and the *B. animalis* BD400 group demonstrate the potential of *B. animalis* BD400 to restore intestinal metabolite disturbance.

The role of these differential metabolites was investigated by annotating the metabolic and regulatory pathways they are associated with. Enrichment results (**Figures 10E, F**) revealed that the differential metabolites primarily belonged to metabolic pathways, suggesting that the CIA model construction significantly impacted the metabolic pathways of rats, and *B. animalis* BD400 also influenced the metabolic pathways of CIA rats. The differential abundance score (DA score) calculations indicated a positive trend for all metabolites in these pathways (**Figure 10G**), whereas in the *B. animalis* BD400 group, metabolites in each pathway exhibited a decreasing trend (**Figure 10H**).

To pinpoint key metabolic pathways highly correlated with metabolite differences, enrichment analysis and topological analysis were conducted. There were significant differences in histidine metabolism, pantothenate and CoA biosynthesis, lysine degradation, steroid hormone biosynthesis, primary bile acid biosynthesis between the CIA model group and the *B. animalis* BD400 group (**Figure 10I**). The differential metabolites were mapped to authoritative metabolite databases such as KEGG and PubChem. After obtaining the matching information of differential metabolites, we searched the pathway database of the corresponding species Rattus norvegicus (rat) and analyzed the metabolic pathway, and obtained **Table 3**. In **Table 4**, the metabolic pathway histidine metabolism has the greatest influence, and urocanic acid is the main metabolic substance involved in the histidine metabolism.

**Table 4:** Analysis of related metabolic pathways and metabolites.

Pathway	Total	Hits	Raw *p*	-ln (*p*)	Holm adjust	FDR	Impact	Hits Cpd
D-Arginine and D-ornithine metabolism	4	1	0.098923	2.3134	1	1	0	D-Arginin
Pantothenate and CoA biosynthesis	15	1	0.32442	1.1257	1	1	0.02041	Pantothenic acid
Histidine metabolism	15	1	0.32442	1.1257	1	1	0.14516	Urocanic acid
Propanoate metabolism	20	1	0.40777	0.89704	1	1	0	2-Hydroxybutyric acid
Lysine degradation	20	1	0.40777	0.89704	1	1	0.05435	N6,N6,N6-Trimethyl-L-lysine
Steroid hormone biosynthesis	70	2	0.54546	0.60612	1	1	0.02803	Estrone
Drug metabolism-other enzymes	30	1	0.54546	0.60595	1	1	0	Isoniazid
Pyrimidine metabolism	41	1	0.66117	0.41374	1	1	0.00709	Cytidine
Primary bile acid biosynthesis	46	1	0.70373	0.35136	1	1	0.02976	Glycocholic acid

Total, the number of all metabolites in that pathway; Hits, the number of differential metabolites hitting the pathway; Raw *p*, the *p*-value obtained by enrichment analysis; -ln (*p*), the p-value takes the negative natural logarithm; Holm adjust, the *p*-value is corrected by multiple hypothesis testing using Holm-Bonferroni method; FDR, the *p*-value corrected by multiple hypothesis testing using the false discovery rate (FDR) method; Impact, topology analysis of the impact factors; Hits Cpd, the name of the differential metabolite that hits the pathway.

We further investigated the expression of metabolites involved in histidine metabolism. **Supplementary Figure S1** is a comparison between the CIA model group and the *B. animalis* BD400 group. In **Supplementary Figure S1**, the expressions of 1-methyl-L-histidine and urocanate were significantly down-regulated. This result also suggests that *B. animalis* BD400 may play a preventive role in rheumatoid arthritis through 1-methyl-L-histidine and urocanate in the histidine metabolic pathway. The KEGG ID and KEDD name of the enzyme involved in the **Supplementary Figure S1** are arranged in **Supplementary Table S1.**

## 4 Discussion

RA is a chronic, symmetrical, systemic, inflammatory autoimmune disease with complex etiology and unclear pathogenesis (23). While there are drugs available to treat RA, there is still no effective treatment. In the early stages, RA presents with joint swelling, pain, and dysfunction. As RA progresses, it is marked by different levels of joint stiffness, bone and skeletal muscle atrophy, and can be highly disabling if not treated regularly (1).

Numerous studies conducted worldwide have demonstrated a strong correlation between RA and gut microbiota, especially in the early stage. For instance, a study in the United States utilized 16S sequencing of stool samples taken from 114 RA patients and healthy individuals, revealing an increase in *Prevotella copri* abundance in RA patients, which was linked to a reduction in *Bacteroides* levels and a decline in beneficial microbes (24). Similarly, a Japanese study involving 17 early RA patients observed a similar trend with an increase in *Prevotella copri* abundance and a decrease in *Bacteroides (* 25). Furthermore, researchers Vaahtovuo et al. from Finland employed 16S rRNA hybridization and DNA staining techniques to analyze the fecal microbiota of 51 early RA patients. A comparison with fibromyalgia patients showed a significant decrease in the levels of bacteria within the *Bifidobacteria* and *Bacteroides*-*Porphyromonas*-*Prevotella* group, *Bacteroides fragilis* subgroup, and *Eubacterium rectale*-*Clostridium coccoides* group among early RA patients (26). Zhang et al. conducted a study in China where they analyzed the oral and gut microbiota of 212 fecal samples from both RA patients and healthy controls using metagenomic shotgun sequencing. Their findings revealed a decrease in *Haemophilus* spp. in the gut, dental, or saliva microbiome of RA patients, while an increase in *Lactobacillus salivarius* was observed in these same microbiomes of RA patients (6). Maeda et al. further explored the connection between gut microbiota and RA by transplanting feces from RA patients into germ-free mice. The mice gradually developed ankle swelling two weeks post-transplant and eventually developed RA (25). Additionally, Zeng et al. successfully treated a 20-year-old woman with refractory RA using fecal microbiota transplantation (FMT). By administering fecal suspensions from healthy eight-year-old donors via colonoscopy, they observed no adverse reactions during or after FMT. Notably, the patient’s rheumatic factors significantly decreased (*p* < 0.05) at 42 days post-FMT, with RA symptoms showing improvement 78 days post-FMT (27). These findings underscore the crucial role of gut microbiota in the pathogenesis and progression of RA.

*Bifidobacterium*, the predominant microbe in the gastrointestinal tract, plays a crucial role in maintaining the balance of gut microbiota and overall host health. Numerous clinical, *in vivo*, and *in vitro* studies have demonstrated the beneficial effects of *Bifidobacterium* on conditions such as inflammatory bowel disease, irritable bowel syndrome, cancer, diarrhea, and lactose intolerance. *Bifidobacterium* exerts its effects by inhibiting the growth of pathogens, preserving gut microbiota balance, and safeguarding intestinal barrier integrity through the reduction of intestinal pH (28). Moreover, *Bifidobacterium* produces a variety of metabolites that are advantageous to the host, including vitamins, polyphenols, conjugated linoleic acid, and short-chain fatty acids. These metabolites also have harmful effects on pathogenic microorganisms, such as organic acids, bacteriocins, and bio-surfactants, thereby impeding the proliferation of harmful microorganisms (29). Additionally, *Bifidobacterium* aids in the breakdown of carbohydrate compounds in the host and from dietary sources through sugar degradation pathways, promoting healthy host metabolism. This feature not only ensures the survival of *Bifidobacterium* in the mammalian intestine but also provides essential nutrients for the host and other intestinal microorganisms through cross-feeding (30). Jeong et al. identified a novel *Bifidobacterium longum* RAPO with therapeutic potential from microbiome analysis of RA patients with varying rheumatoid factor (RF) levels. Oral administration of *B. longum* RAPO significantly reduced RA incidence, arthritis score, inflammation, bone injury, cartilage injury, Th17 cells, and inflammatory cytokine secretion in CIA mice (31). Furthermore, patents suggest that certain *Bifidobacterium* strains may have preventive or therapeutic effects on RA (32–35). This study demonstrated that oral administration of *B. animalis* BD400 for nine weeks notably decreased arthritis clinical scores and paw thickness in CIA rats (**Figures 2I–K**). Histological analysis of knee joint sections and staining of rats revealed that *B. animalis* BD400 improved the interface between synovium and bone tissue, reduced cell infiltration and synovial hyperplasia, restored the thickness and integrity of the hyaline cartilage layer, ultimately alleviating RA symptoms (**Figures 3**, **4**).

The impact of *B. animalis* BD400 on RA symptoms prompted further investigation into its effects on specific antibodies and pro-inflammatory factors in the serum and joint fluid of CIA rats. Collagen-induced arthritis, a well-established model for RA, shares key characteristics with human RA, notably immune tolerance breakdown and autoantibody production (13). Autoantibodies, particularly anti-CII IgG antibodies specific to type II collagen, play a crucial role in RA diagnosis and disease progression. High levels of these autoantibodies can be detected in serum well before clinical symptoms manifest (19). In this study, anti-CII IgG and its subtypes (IgG1, IgG2a, and IgG2b) were measured in the serum of CIA rats. Levels of these antibodies were significantly elevated in CIA rats compared to the control group. Treatment with *B. longum* BD3150 and *B. animalis* BD400 led to a significant decrease in anti-CII IgG, IgG1, and IgG2a levels (*p* < 0.05) (**Figure 5**). Meanwhile, we assessed the production of pro-inflammatory factors, including IL-17A, TNF-α, and IL-1β, in both serum and synovial fluid. The levels of IL-17A in serum, as well as TNF-α and IL-1β in synovial fluid, were significantly reduced with the administration of *B. longum* BD3150 and *B. animalis* BD400. These inflammatory factors play a crucial role in cellular communication. Notably, TNF-α, IL-6, and IL-1β are commonly studied in the context of RA. TNF-α, a member of the tumor necrosis factor family, triggers the production of various cytokines and proteases by target cells in RA, primarily through the NF-κB and MAPK signaling pathways (36). This cascade of inflammatory responses leads to a continuous cycle of inflammation, contributing to bone and joint erosion (37). As a result, the components of the articular cavity, such as synovial cells, synovial tissue fluid, and serum in CIA rats, exhibited an increasing trend compared to healthy controls. The intervention of *B. longum* BD3150 and *B. animalis* BD400 reduced the levels of TNF-α in the synovial fluid of CIA rats, suggesting the disruption of this persistent inflammatory cycle. In RA, antigen-presenting cells carrying HLA-DR antigen increase and stimulate CD4+T lymphocytes to secrete numerous cytokines, activating synovial macrophages to release IL-1β and TNF⁃α (38). These cytokines target joint cells, leading to the production of collagen and neutral protease, ultimately causing synovial proliferation and erosion of joint cartilage, worsening the disease (38). While IL⁃1β levels are typically low in the knee fluid of healthy individuals, our study found elevated levels of IL-1β in the joint fluid of collagen-induced arthritis (CIA) rats. Treatment with B. longum BD3150 and B. animalis BD400 reduced IL-1β levels in the knee fluid of CIA rats. IL-17A, a key inflammatory mediator in RA, is primarily produced by CD4+Th17 cells, as well as CD8+T cells, NK T cells, γδT cells, neutrophils, and lymphoid tissue inductor-like cells (39). IL-17A works in conjunction with TNF-α to promote osteogenesis, induce PEG production in chondrocytes, stimulate fibroblast-like synoviocytes (FLS) to secrete various inflammatory factors, and activate pathways like PI3K/Akt and NF⁃κB, leading to synovial inflammation and cartilage damage (40). Consequently, IL⁃17A levels are higher in the synovial fluid and surrounding tissues of RA patients compared to healthy individuals. Our study demonstrated a significant decrease in serum IL-17A levels in CIA rats following the oral administration of probiotics, highlighting the potential of *B. longum* BD3150 and *B. animalis* BD400 in mitigating RA-related inflammation.

The occurrence and development of RA is closely linked to disruptions in both local and systemic immune responses, stemming from immune sites beyond the joints, known as the “mucosal origin hypothesis” (41–43). The gut, recognized as the largest immune organ, harbors a diverse population of microorganisms that not only provide essential nutrients and energy to the body but also play a role in shaping and regulating the intestinal mucosal immune system (44). These intestinal microorganisms interact with, respond to, and regulate the intestinal mucosa through the intestinal mucosal barrier (45). The tight connection between the mucus barrier and intestinal epithelial cells serves as the primary defense against pathogens (46). Key proteins involved in maintaining the integrity of the intestinal barrier include claudin-1, occludin-1, mucin-2 (MUC-2), and zonula occludens-1 (ZO-1). In a study involving collagen-induced arthritis (CIA) rats, it was found that the expression of occludin-1 and MUC-2 in the gut was significantly reduced compared to control rats (*p* < 0.001), while the expression of claudin-1 was significantly increased (*p* < 0.001). Treatment with 5 strains of *Bifidobacterium* (BD3150, BD400, BD6256, BD5348, BB12) restored the expression of these three proteins (claudin-1, occludin-1, and MUC-2) to normal levels (**Figure 6**). The relationship between gut microbiota and human health is integral, as gut microbiota are present throughout the human life cycle. Changes in gut microbiota composition result from specific interactions between microorganisms and the gut environment. The evolution and selection process of the gut microbiota remains unclear, but there is growing interest in the co-evolution and mutual interaction between the gut microbiota and the host intestinal immune system. The intestinal microbiota plays a crucial role in supporting the development of the immune system through various mechanisms and helps maintain a balanced intestinal micro-ecology (47, 48). Imbalances in the intestinal micro-ecology have been linked to various diseases, including intestinal and immune disorders (49, 50). This imbalance can lead to the disruption of the intestinal micro-ecology, an increase in pathogenic bacteria, a decrease in beneficial bacteria, triggering local inflammatory responses and cascading effects that impact the immune response of extra-intestinal organs, ultimately posing a threat to the host’s health (51). In our study (**Figures 7**–**9**), significant differences were observed in the gut microbiota composition between CIA rats and normal rats (*p* < 0.05). *Desulfobacterota* was identified as the phylum with the most significant difference between the CIA model group and the control group (*p* < 0.05). Treatment with *B. breve* BB12 led to a restoration in the relative abundance of *Desulfobacterota*. At the genus level, notable differences were observed in *Clostridia_*UCG-014_unclassified, *Lactobacillus*, *Ligilactobacillus*, *Lachnospiraceae*_unclassified, and *Firmicutes*_unclassified (top 5 species). The combined proportion of these top 5 species in the CIA model group was 50.72%, significantly different from the control group’s 34.57%. The percentages of these top 5 species decreased under the administration of *B. bifidum* BD5348. Through differential abundance analysis, we observed that *B. animalis* BD400 decreased the relative abundance of HT002. *Desulfobacterota*, a sulfate-reducing anaerobic bacteria, thrives in the gut and releases hydrogen sulfide. The role of hydrogen sulfide is intricate and at times contradictory. Clinical data suggests an association between hydrogen sulfide and chronic colon disease as well as inflammation of the large intestine (52). However, research has also demonstrated that hydrogen sulfide can directly stimulate angiogenesis, crucial for mending gastrointestinal ulcers (53, 54). Despite the challenging task of elucidating the precise role of *Desulfobacterota* in the gut, our findings revealed contrasting results in the abundance of *Desulfobacterota* between the CIA model group and the *B. animalis* BD400 group, providing insight into the ability of *B. animalis* BD400 to restore gut microbiota. Furthermore, a separate study on *Bifidobacterium adolescentis* yielded similar biological outcomes, highlighting *Lactobacillus*, *Ligilactobacillus*, and *Lachnospiraceae* as prominent genera (55). This finding strongly corroborates our own results.

The metabolites of gut microbiota can directly or indirectly impact host health. These metabolites can be categorized based on their synthesis pathway:

1. Metabolites produced by gut microbiota that break down dietary components like short-chain fatty acids, tryptophan, and trimethylamine oxide; 2. Metabolites produced by the host and altered by gut microbiota, such as bile acids; 3. Metabolites synthesized by gut microbiota from scratch, including branched-chain amino acids, polyamines, and vitamins (56). These gut microbiota metabolites play a crucial role in maintaining host intestinal balance and regulating immune function through various mechanisms. They can enter cells through passive diffusion or carrier-mediated transport to regulate processes like protein synthesis, glucose and lipid metabolism, insulin resistance, hepatocyte proliferation, and immunity. Moreover, these metabolites can travel to distant tissues and organs via the bloodstream, influencing the functions of multiple organs (57–60). In this study (**Figure 10**), metabolites including crotonoside, guanosine, 2`-ketobutyric acid, momordicinin, botulin, trigonelline and so on showed a significant difference in CIA rats. After oral *B. animalis* BD400, these differences are restored, especially 2`-deoxyuridine, 2`-ketobutyric acid, crotonoside and guanosine. These results also suggest that the role of *B. animalis* BD400 in protecting from RA is achieved through the regulation of gut microbiota metabolites. So we conducted enrichment and topological analyses to find the key pathways which is the highest correlation to these metabolites. The expression of 1-methyl-L-histidine and urocanate in histidine metabolism was down-regulated in gut microbiota in CIA rats given *B. animalis* BD400 orally (**Supplementary Figure S1**). Amino acids are energy sources and substrates for cellular protein and nucleic acid biosynthesis, and a variety of amino acids and their transporters are necessary for T cell activation, differentiation and effector function (61). Histidine, lysine and threonine can inhibit the mTOR signaling pathway and IgE mediated mast cell activation (62). Studies found that lack of histidine or arginine, TGF-β1 extracellular concentration and TGF-β1 mRNA levels decreased, intestinal epithelial cell repair significantly decreased. However, supplementation with 10 µM histidine or 50 µM arginine restored cell repair and TGF-β1 extracellular concentrations. This suggests that the absence of histidine or arginine affects IEC repair by reducing TGF-β1 (63). In our study, reduction in the levels of 1-methyl-L-histidine and urocanate in histidine metabolism leads to the decrease of inflammatory response, highlighting its role in *B. animalis* BD400 regulation. These results indicate that *B. animalis* BD400 protect from RA through the regulation of 1-methyl-L-histidine and urocanate in histidine metabolism. The main objective of this study was to screen out a probiotic strain, *B. animalis* BD400, which has a protective effect on RA. But the key target for *B. animalis* BD400 to play a role is what we need to study next. As mentioned above, 1-methyl-L-histidine and urocanate in histidine metabolism are significantly down-regulated with the intervention of *B. animalis* BD400, but the key targets are still unclear. This will also be the key point of our next study.

## 5 Conclusion

The clinical treatment of RA focuses on reducing inflammation and alleviating symptoms, as RA is challenging to cure. In our study, the expression of 1-methyl-L-histidine and urocanate in histidine metabolism was reduced by early administration of *B. animalis* BD400, thus achieving the purpose of protecting from RA. This research is expected to provide a basis for the daily prevention of RA.

## Data Availability

The raw sequence data of 16S rDNA gene sequencing were deposited in the Sequence Read Archive (SRA) at NCBI under Bioproject PRJNA1139339 (SUB14619059, https://www.ncbi.nlm.nih.gov/bioproject/PRJNA1139339). The data matrix for non-targeted metabolite analysis was supplied in **Supplementary Table S2.**
