# Supplementary figures and images for: Bifidobacterium animalis BD400 protects from collagen-induced arthritis through histidine metabolism
Source: Front Immunol. 2025 Jan 22;16:1518181. doi: 10.3389/fimmu.2025.1518181 (PMC11794514; doi:10.3389/fimmu.2025.1518181)

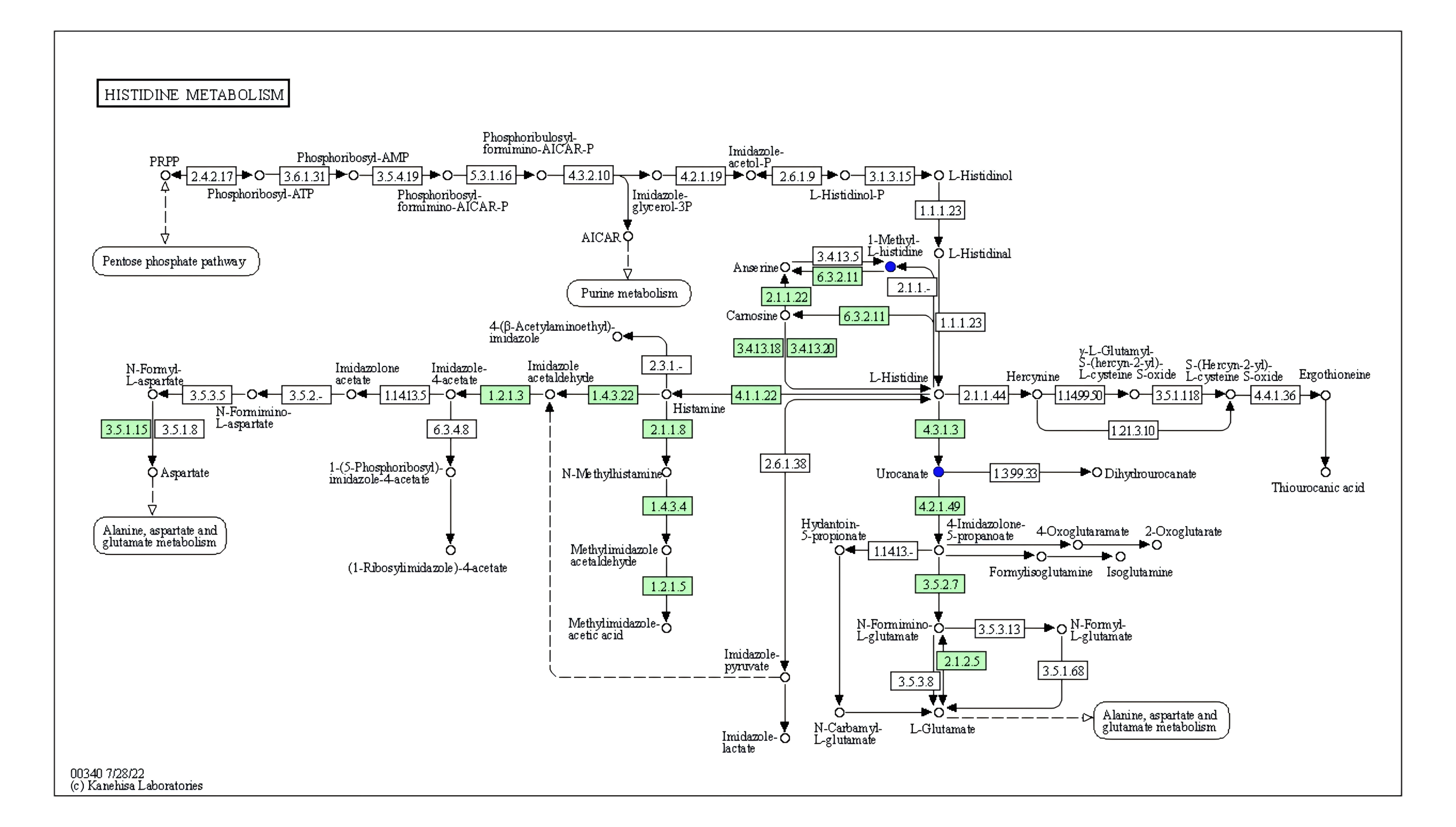

Supplement: Supplementary file 1 [file Image1.jpeg]

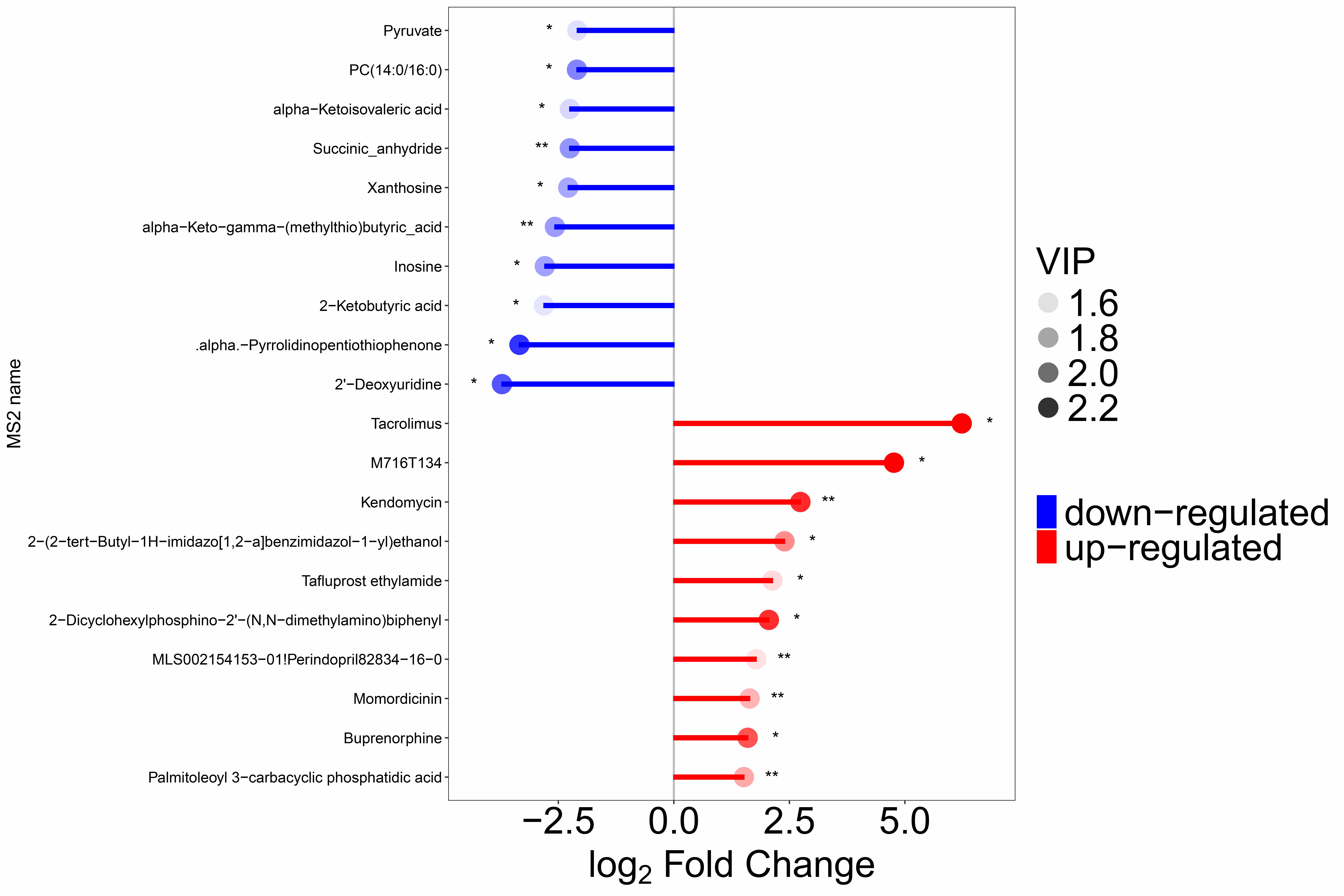

Supplement: Supplementary file 3 [file Image3.jpeg]

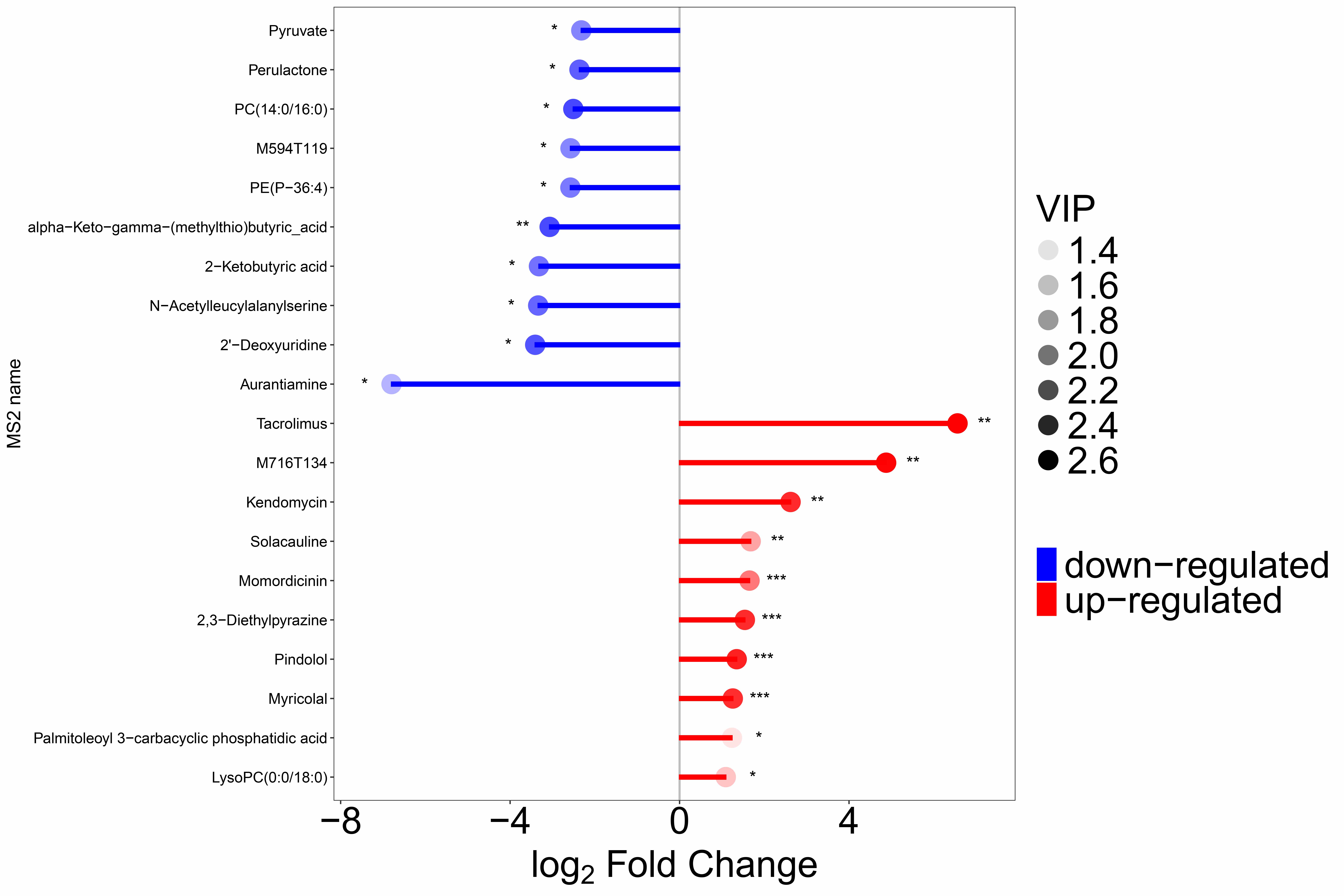

Supplement: Supplementary file 5 [file Image5.jpeg]
