# Supplementary material for: Bifidobacterium animalis BD400 protects from collagen-induced arthritis through histidine metabolism
Source: Front Immunol. 2025 Jan 22;16:1518181. doi: 10.3389/fimmu.2025.1518181 (PMC11794514; doi:10.3389/fimmu.2025.1518181)
Supplement: Supplementary file 8 [file Table3.docx]

**1.Figure 4**

**TNF-a**

| **ANOVA** | | | | | |
| --- | --- | --- | --- | --- | --- |
| Area | | | | | |
|  | sum of squares | df | mean square | F | significance |
| interblock | 17.110 | 7 | 2.444 | 349.032 | .000 |
| intra-class | .336 | 48 | .007 |  |  |
| sum | 17.446 | 55 |  |  |  |

| **multiple comparisons** | | | | | | | |
| --- | --- | --- | --- | --- | --- | --- | --- |
| Area | | | | | | | |
|  | (I) group | (J) group | Mean Difference  (I-J) | standard error | significance | 95% confidence interval | |
|  |  |  |  |  |  | lower limit | upper limit |
| Tukey HSD | 8.Control | 1.BD3150 | -.421403385143^*^ | .044730516932 | .000 | -.56312240670 | -.27968436359 |
|  |  | 2.BD400 | -.131113223429 | .044730516932 | .089 | -.27283224498 | .01060579812 |
|  |  | 3.BD6256 | -1.246702873000^*^ | .044730516932 | .000 | -1.38842189455 | -1.10498385145 |
|  |  | 4.BD5348 | -1.195150434143^*^ | .044730516932 | .000 | -1.33686945570 | -1.05343141259 |
|  |  | 5.BB12 | -.542202277286^*^ | .044730516932 | .000 | -.68392129884 | -.40048325573 |
|  |  | 6.MTX | -.806999022714^*^ | .044730516932 | .000 | -.94871804427 | -.66528000116 |
|  |  | 7.Model | -1.698498221429^*^ | .044730516932 | .000 | -1.84021724298 | -1.55677919988 |
| *. The significance level of mean difference was 0.05. | | | | | | | |

**MMP-13**

| **ANOVA** | | | | | |
| --- | --- | --- | --- | --- | --- |
| Area | | | | | |
|  | sum of squares | df | mean square | F | significance |
| interblock | 145.621 | 7 | 20.803 | 4377.216 | .000 |
| intra-class | .228 | 48 | .005 |  |  |
| sum | 145.849 | 55 |  |  |  |

| **multiple comparisons** | | | | | | | |
| --- | --- | --- | --- | --- | --- | --- | --- |
| Area | | | | | | | |
|  | (I) group | (J) group | Mean Difference  (I-J) | standard error | significance | 95% confidence interval | |
|  |  |  |  |  |  | lower limit | upper limit |
| Tukey HSD | 8.Control | 1.BD3150 | -1.729337837571^*^ | .036849318309 | .000 | -1.84608697650 | -1.61258869864 |
|  |  | 2.BD400 | -.773780423429^*^ | .036849318309 | .000 | -.89052956236 | -.65703128450 |
|  |  | 3.BD6256 | -2.460078550429^*^ | .036849318309 | .000 | -2.57682768936 | -2.34332941150 |
|  |  | 4.BD5348 | -3.294947883857^*^ | .036849318309 | .000 | -3.41169702279 | -3.17819874493 |
|  |  | 5.BB12 | -2.063783910143^*^ | .036849318309 | .000 | -2.18053304907 | -1.94703477121 |
|  |  | 6.MTX | -2.093230559143^*^ | .036849318309 | .000 | -2.20997969807 | -1.97648142021 |
|  |  | 7.Model | -5.728131582429^*^ | .036849318309 | .000 | -5.84488072136 | -5.61138244350 |
| *. The significance level of mean difference was 0.05. | | | | | | | |

**2.Figure 5**

**Anti-CII IgG**

| **ANOVA** | | | | | |
| --- | --- | --- | --- | --- | --- |
| value | | | | | |
|  | sum of squares | df | mean square | F | significance |
| interblock | 1801.437 | 7 | 257.348 | 1.150 | .346 |
| intra-class | 12080.268 | 54 | 223.709 |  |  |
| sum | 13881.705 | 61 |  |  |  |

| **multiple comparisons** | | | | | | | |
| --- | --- | --- | --- | --- | --- | --- | --- |
| value | | | | | | | |
|  | (I) group | (J) group | Mean Difference  (I-J) | standard error | significance | 95% confidence interval | |
|  |  |  |  |  |  | lower limit | upper limit |
| Tukey HSD | 7.Model | 1.BD3150 | 5.88334 | 7.47845 | .993 | -17.6941 | 29.4608 |
|  |  | 2.BD400 | 10.39089 | 7.47845 | .858 | -13.1865 | 33.9683 |
|  |  | 3.BD6256 | 11.76300 | 7.47845 | .764 | -11.8144 | 35.3404 |
|  |  | 4.BD5348 | -.20701 | 7.47845 | 1.000 | -23.7844 | 23.3704 |
|  |  | 5.BB12 | 4.87431 | 7.47845 | .998 | -18.7031 | 28.4517 |
|  |  | 6.MTX | -.04078 | 8.07765 | 1.000 | -25.5073 | 25.4257 |
|  |  | 8.Control | -4.65822 | 7.47845 | .998 | -28.2356 | 18.9192 |
|  | 8.Control | 1.BD3150 | 10.54156 | 7.47845 | .849 | -13.0359 | 34.1190 |
|  |  | 2.BD400 | 15.04911 | 7.47845 | .483 | -8.5283 | 38.6265 |
|  |  | 3.BD6256 | 16.42122 | 7.47845 | .370 | -7.1562 | 39.9986 |
|  |  | 4.BD5348 | 4.45121 | 7.47845 | .999 | -19.1262 | 28.0286 |
|  |  | 5.BB12 | 9.53253 | 7.47845 | .904 | -14.0449 | 33.1100 |
|  |  | 6.MTX | 4.61744 | 8.07765 | .999 | -20.8491 | 30.0840 |
|  |  | 7.Model | 4.65822 | 7.47845 | .998 | -18.9192 | 28.2356 |

**Anti-CII IgG1**

| **ANOVA** | | | | | |
| --- | --- | --- | --- | --- | --- |
| value | | | | | |
|  | sum of squares | df | mean square | F | significance |
| interblock | 3035.840 | 7 | 433.691 | 8.501 | .000 |
| intra-class | 2754.798 | 54 | 51.015 |  |  |
| sum | 5790.638 | 61 |  |  |  |

| **多重比较** | | | | | | | |
| --- | --- | --- | --- | --- | --- | --- | --- |
| Area | | | | | | | |
|  | (I)group | (J) group | Mean Difference  (I-J) | standard error | significance | 95% confidence interval | |
|  |  |  |  |  |  | lower limit | upper limit |
| Tukey HSD | 7.Model | 1.BD3150 | 2.76612 | 3.57123 | .994 | -8.4930 | 14.0252 |
|  |  | 2.BD400 | 9.84741 | 3.57123 | .128 | -1.4117 | 21.1065 |
|  |  | 3.BD6256 | 4.35375 | 3.57123 | .923 | -6.9053 | 15.6128 |
|  |  | 4.BD5348 | 5.51676 | 3.57123 | .780 | -5.7423 | 16.7758 |
|  |  | 5.BB12 | 2.93715 | 3.57123 | .991 | -8.3219 | 14.1962 |
|  |  | 6.MTX | -1.44521 | 3.85737 | 1.000 | -13.6064 | 10.7160 |
|  |  | 8.Control | -15.04695^*^ | 3.57123 | .002 | -26.3060 | -3.7879 |
|  | 8.Control | 1.BD3150 | 17.81308^*^ | 3.57123 | .000 | 6.5540 | 29.0722 |
|  |  | 2.BD400 | 24.89437^*^ | 3.57123 | .000 | 13.6353 | 36.1534 |
|  |  | 3.BD6256 | 19.40070^*^ | 3.57123 | .000 | 8.1416 | 30.6598 |
|  |  | 4.BD5348 | 20.56371^*^ | 3.57123 | .000 | 9.3046 | 31.8228 |
|  |  | 5.BB12 | 17.98411^*^ | 3.57123 | .000 | 6.7250 | 29.2432 |
|  |  | 6.MTX | 13.60174^*^ | 3.85737 | .018 | 1.4405 | 25.7629 |
|  |  | 7.Model | 15.04695^*^ | 3.57123 | .002 | 3.7879 | 26.3060 |
| *. The significance level of mean difference was 0.05. | | | | | | | |

**Anti-CII IgG2a**

| **ANOVA** | | | | | |
| --- | --- | --- | --- | --- | --- |
| value | | | | | |
|  | sum of squares | df | mean square | F | significance |
| interblock | 847.444 | 7 | 121.063 | 9.819 | .000 |
| intra-class | 665.784 | 54 | 12.329 |  |  |
| sum | 1513.228 | 61 |  |  |  |

| **multiple comparisons** | | | | | | | |
| --- | --- | --- | --- | --- | --- | --- | --- |
| value | | | | | | | |
|  | (I) group | (J) group | Mean Difference  (I-J) | standard error | significance | 95% confidence interval | |
|  |  |  |  |  |  | lower limit | upper limit |
| Tukey HSD | 7.Model | 1.BD3150 | 6.28164^*^ | 1.75566 | .016 | .7465 | 11.8167 |
|  |  | 2.BD400 | 5.78288^*^ | 1.75566 | .035 | .2478 | 11.3180 |
|  |  | 3.BD6256 | 3.06367 | 1.75566 | .658 | -2.4714 | 8.5988 |
|  |  | 4.BD5348 | 2.69928 | 1.75566 | .784 | -2.8358 | 8.2344 |
|  |  | 5.BB12 | 1.95740 | 1.75566 | .951 | -3.5777 | 7.4925 |
|  |  | 6.MTX | -.49370 | 1.89633 | 1.000 | -6.4723 | 5.4849 |
|  |  | 8.Control | -5.97168^*^ | 1.75566 | .026 | -11.5068 | -.4366 |
|  | 8.Control | 1.BD3150 | 12.25332^*^ | 1.75566 | .000 | 6.7182 | 17.7884 |
|  |  | 2.BD400 | 11.75457^*^ | 1.75566 | .000 | 6.2195 | 17.2897 |
|  |  | 3.BD6256 | 9.03535^*^ | 1.75566 | .000 | 3.5003 | 14.5704 |
|  |  | 4.BD5348 | 8.67097^*^ | 1.75566 | .000 | 3.1359 | 14.2061 |
|  |  | 5.BB12 | 7.92908^*^ | 1.75566 | .001 | 2.3940 | 13.4642 |
|  |  | 6.MTX | 5.47798 | 1.89633 | .095 | -.5006 | 11.4566 |
|  |  | 7.Model | 5.97168^*^ | 1.75566 | .026 | .4366 | 11.5068 |
| *. The significance level of mean difference was 0.05. | | | | | | | |

**Anti-CII IgG2b**

| **ANOVA** | | | | | |
| --- | --- | --- | --- | --- | --- |
| value | | | | | |
|  | sum of squares | df | mean square | F | significance |
| interblock | 189.028 | 7 | 27.004 | 4.741 | .000 |
| intra-class | 307.576 | 54 | 5.696 |  |  |
| sum | 496.603 | 61 |  |  |  |

| **multiple comparisons** | | | | | | | |
| --- | --- | --- | --- | --- | --- | --- | --- |
| value | | | | | | | |
|  | (I) group | (J) group | Mean Difference  (I-J) | standard error | significance | 95% confidence interval | |
|  |  |  |  |  |  | lower limit | upper limit |
| Tukey HSD | 7.Model | 1.BD3150 | 3.30762 | 1.19330 | .124 | -.4545 | 7.0698 |
|  |  | 2.BD400 | -1.15657 | 1.19330 | .977 | -4.9187 | 2.6056 |
|  |  | 3.BD6256 | 1.27821 | 1.19330 | .960 | -2.4839 | 5.0403 |
|  |  | 4.BD5348 | 2.06466 | 1.19330 | .668 | -1.6975 | 5.8268 |
|  |  | 5.BB12 | -.67938 | 1.19330 | .999 | -4.4415 | 3.0827 |
|  |  | 6.MTX | 1.85057 | 1.28891 | .837 | -2.2130 | 5.9141 |
|  |  | 8.Control | -2.13506 | 1.19330 | .630 | -5.8972 | 1.6271 |
|  | 8.Control | 1.BD3150 | 5.44268^*^ | 1.19330 | .001 | 1.6805 | 9.2048 |
|  |  | 2.BD400 | .97849 | 1.19330 | .991 | -2.7836 | 4.7406 |
|  |  | 3.BD6256 | 3.41327 | 1.19330 | .102 | -.3489 | 7.1754 |
|  |  | 4.BD5348 | 4.19972^*^ | 1.19330 | .019 | .4376 | 7.9618 |
|  |  | 5.BB12 | 1.45567 | 1.19330 | .922 | -2.3065 | 5.2178 |
|  |  | 6.MTX | 3.98563 | 1.28891 | .058 | -.0779 | 8.0492 |
|  |  | 7.Model | 2.13506 | 1.19330 | .630 | -1.6271 | 5.8972 |
| *. The significance level of mean difference was 0.05. | | | | | | | |

**IL-17A**

| **ANOVA** | | | | | |
| --- | --- | --- | --- | --- | --- |
| value | | | | | |
|  | sum of squares | df | mean square | F | significance |
| interblock | 181.623 | 7 | 25.946 | 1.911 | .086 |
| intra-class | 733.292 | 54 | 13.579 |  |  |
| sum | 914.916 | 61 |  |  |  |

| **multiple comparisons** | | | | | | | |
| --- | --- | --- | --- | --- | --- | --- | --- |
| value | | | | | | | |
|  | (I) group | (J) group | Mean Difference  (I-J) | standard error | significance | 95% confidence interval | |
|  |  |  |  |  |  | lower limit | upper limit |
| Tukey HSD | 7.Model | 1.BD3150 | -3.26554 | 1.84252 | .641 | -9.0745 | 2.5434 |
|  |  | 2.BD400 | -2.49724 | 1.84252 | .873 | -8.3062 | 3.3117 |
|  |  | 3.BD6256 | -2.99126 | 1.84252 | .734 | -8.8002 | 2.8177 |
|  |  | 4.BD5348 | -3.29285 | 1.84252 | .631 | -9.1018 | 2.5161 |
|  |  | 5.BB12 | -2.63464 | 1.84252 | .839 | -8.4436 | 3.1743 |
|  |  | 6.MTX | 1.44532 | 1.99015 | .996 | -4.8290 | 7.7197 |
|  |  | 8.Control | .35648 | 1.84252 | 1.000 | -5.4525 | 6.1654 |
|  | 8.Control | 1.BD3150 | -3.62202 | 1.84252 | .514 | -9.4310 | 2.1869 |
|  |  | 2.BD400 | -2.85372 | 1.84252 | .778 | -8.6627 | 2.9552 |
|  |  | 3.BD6256 | -3.34774 | 1.84252 | .612 | -9.1567 | 2.4612 |
|  |  | 4.BD5348 | -3.64933 | 1.84252 | .504 | -9.4583 | 2.1596 |
|  |  | 5.BB12 | -2.99112 | 1.84252 | .734 | -8.8001 | 2.8178 |
|  |  | 6.MTX | 1.08884 | 1.99015 | .999 | -5.1855 | 7.3632 |
|  |  | 7.Model | -.35648 | 1.84252 | 1.000 | -6.1654 | 5.4525 |

**IL-1β**

| **ANOVA** | | | | | |
| --- | --- | --- | --- | --- | --- |
| value | | | | | |
|  | sum of squares | df | mean square | F | significance |
| interblock | 4838.021 | 7 | 691.146 | 1.173 | .334 |
| intra-class | 31821.543 | 54 | 589.288 |  |  |
| sum | 36659.564 | 61 |  |  |  |

| **multiple comparisons** | | | | | | | |
| --- | --- | --- | --- | --- | --- | --- | --- |
| value | | | | | | | |
|  | (I) group | (J) group | Mean Difference  (I-J) | standard error | significance | 95% confidence interval | |
|  |  |  |  |  |  | lower limit | upper limit |
| Tukey HSD | 7.Model | 1.BD3150 | 3.68554 | 12.13763 | 1.000 | -34.5810 | 41.9520 |
|  |  | 2.BD400 | 21.13408 | 12.13763 | .661 | -17.1324 | 59.4006 |
|  |  | 3.BD6256 | 21.49491 | 12.13763 | .642 | -16.7716 | 59.7614 |
|  |  | 4.BD5348 | 10.16272 | 12.13763 | .990 | -28.1038 | 48.4292 |
|  |  | 5.BB12 | 4.20140 | 12.13763 | 1.000 | -34.0651 | 42.4679 |
|  |  | 6.MTX | 26.24413 | 13.11013 | .490 | -15.0884 | 67.5767 |
|  |  | 8.Control | 13.31596 | 12.13763 | .955 | -24.9505 | 51.5825 |
|  | 8.Control | 1.BD3150 | -9.63042 | 12.13763 | .993 | -47.8969 | 28.6361 |
|  |  | 2.BD400 | 7.81812 | 12.13763 | .998 | -30.4484 | 46.0846 |
|  |  | 3.BD6256 | 8.17895 | 12.13763 | .997 | -30.0875 | 46.4454 |
|  |  | 4.BD5348 | -3.15323 | 12.13763 | 1.000 | -41.4197 | 35.1133 |
|  |  | 5.BB12 | -9.11456 | 12.13763 | .995 | -47.3811 | 29.1519 |
|  |  | 6.MTX | 12.92817 | 13.11013 | .975 | -28.4044 | 54.2607 |
|  |  | 7.Model | -13.31596 | 12.13763 | .955 | -51.5825 | 24.9505 |

**TNF-α**

| **ANOVA** | | | | | |
| --- | --- | --- | --- | --- | --- |
| value | | | | | |
|  | sum of squares | df | mean square | F | significance |
| interblock | 778.910 | 7 | 111.273 | .305 | .949 |
| intra-class | 19684.962 | 54 | 364.536 |  |  |
| sum | 20463.872 | 61 |  |  |  |

| **multiple comparisons** | | | | | | | |
| --- | --- | --- | --- | --- | --- | --- | --- |
| value | | | | | | | |
|  | (I) group | (J) group | Mean Difference  (I-J) | standard error | significance | 95% confidence interval | |
|  |  |  |  |  |  | lower limit | upper limit |
| Tukey HSD | 7.Model | 1.BD3150 | 12.46410 | 9.54642 | .893 | -17.6331 | 42.5612 |
|  |  | 2.BD400 | 2.27276 | 9.54642 | 1.000 | -27.8244 | 32.3699 |
|  |  | 3.BD6256 | 2.60500 | 9.54642 | 1.000 | -27.4921 | 32.7021 |
|  |  | 4.BD5348 | 4.64024 | 9.54642 | 1.000 | -25.4569 | 34.7374 |
|  |  | 5.BB12 | 4.70950 | 9.54642 | 1.000 | -25.3877 | 34.8066 |
|  |  | 6.MTX | 6.71496 | 10.31131 | .998 | -25.7937 | 39.2236 |
|  |  | 8.Control | 3.31849 | 9.54642 | 1.000 | -26.7787 | 33.4156 |
|  | 8.Control | 1.BD3150 | 9.14561 | 9.54642 | .978 | -20.9515 | 39.2428 |
|  |  | 2.BD400 | -1.04574 | 9.54642 | 1.000 | -31.1429 | 29.0514 |
|  |  | 3.BD6256 | -.71349 | 9.54642 | 1.000 | -30.8106 | 29.3837 |
|  |  | 4.BD5348 | 1.32174 | 9.54642 | 1.000 | -28.7754 | 31.4189 |
|  |  | 5.BB12 | 1.39101 | 9.54642 | 1.000 | -28.7061 | 31.4882 |
|  |  | 6.MTX | 3.39647 | 10.31131 | 1.000 | -29.1122 | 35.9051 |
|  |  | 7.Model | -3.31849 | 9.54642 | 1.000 | -33.4156 | 26.7787 |

**3.Figure 6**

**Claudin-1**

| **ANOVA** | | | | | |
| --- | --- | --- | --- | --- | --- |
| value | | | | | |
|  | sum of squares | df | mean square | F | significance |
| interblock | 120.277 | 7 | 17.182 | 6.737 | .000 |
| intra-class | 137.723 | 54 | 2.550 |  |  |
| sum | 258.000 | 61 |  |  |  |

| **multiple comparisons** | | | | | | | |
| --- | --- | --- | --- | --- | --- | --- | --- |
| value | | | | | | | |
|  | (I) group | (J) group | Mean Difference  (I-J) | standard error | significance | 95% confidence interval | |
|  |  |  |  |  |  | lower limit | upper limit |
| Tukey HSD | 7.Model | 1.BD3150 | 3.14375^*^ | .79850 | .005 | .6263 | 5.6612 |
|  |  | 2.BD400 | 4.44250^*^ | .79850 | .000 | 1.9250 | 6.9600 |
|  |  | 3.BD6256 | 1.59750 | .79850 | .491 | -.9200 | 4.1150 |
|  |  | 4.BD5348 | 2.59250^*^ | .79850 | .039 | .0750 | 5.1100 |
|  |  | 5.BB12 | 3.40875^*^ | .79850 | .002 | .8913 | 5.9262 |
|  |  | 6.MTX | 3.95333^*^ | .86248 | .001 | 1.2342 | 6.6725 |
|  |  | 8.Control | 4.04625^*^ | .79850 | .000 | 1.5288 | 6.5637 |
| *. The significance level of mean difference was 0.05. | | | | | | | |

**MUC-2**

| **ANOVA** | | | | | |
| --- | --- | --- | --- | --- | --- |
| value | | | | | |
|  | sum of squares | df | mean square | F | significance |
| interblock | 46.650 | 7 | 6.664 | 11.183 | .000 |
| intra-class | 32.181 | 54 | .596 |  |  |
| sum | 78.832 | 61 |  |  |  |

| **multiple comparisons** | | | | | | | |
| --- | --- | --- | --- | --- | --- | --- | --- |
| value | | | | | | | |
|  | (I) group | (J) group | Mean Difference  (I-J) | standard error | significance | 95% confidence interval | |
|  |  |  |  |  |  | lower limit | upper limit |
| Tukey HSD | 7.Model | 1.BD3150 | -1.94500^*^ | .38599 | .000 | -3.1619 | -.7281 |
|  |  | 2.BD400 | -2.45625^*^ | .38599 | .000 | -3.6732 | -1.2393 |
|  |  | 3.BD6256 | -1.66500^*^ | .38599 | .002 | -2.8819 | -.4481 |
|  |  | 4.BD5348 | -1.64750^*^ | .38599 | .002 | -2.8644 | -.4306 |
|  |  | 5.BB12 | -1.41750^*^ | .38599 | .012 | -2.6344 | -.2006 |
|  |  | 6.MTX | -2.40625^*^ | .41692 | .000 | -3.7207 | -1.0918 |
|  |  | 8.Control | -3.09500^*^ | .38599 | .000 | -4.3119 | -1.8781 |
| *. The significance level of mean difference was 0.05. | | | | | | | |

**Occludin-1**

| **ANOVA** | | | | | |
| --- | --- | --- | --- | --- | --- |
| value | | | | | |
|  | sum of squares | df | mean square | F | significance |
| interblock | 202.698 | 7 | 28.957 | 99.025 | .000 |
| intra-class | 15.791 | 54 | .292 |  |  |
| sum | 218.489 | 61 |  |  |  |

| **multiple comparisons** | | | | | | | |
| --- | --- | --- | --- | --- | --- | --- | --- |
| value | | | | | | | |
|  | (I) group | (J) group | Mean Difference  (I-J) | standard error | significance | 95% confidence interval | |
|  |  |  |  |  |  | lower limit | upper limit |
| Tukey HSD | 7.Model | 1.BD3150 | -2.14250^*^ | .27038 | .000 | -2.9949 | -1.2901 |
|  |  | 2.BD400 | -3.84375^*^ | .27038 | .000 | -4.6962 | -2.9913 |
|  |  | 3.BD6256 | -1.16250^*^ | .27038 | .002 | -2.0149 | -.3101 |
|  |  | 4.BD5348 | -1.33375^*^ | .27038 | .000 | -2.1862 | -.4813 |
|  |  | 5.BB12 | -1.01875^*^ | .27038 | .009 | -1.8712 | -.1663 |
|  |  | 6.MTX | -4.83625^*^ | .29204 | .000 | -5.7570 | -3.9155 |
|  |  | 8.Control | -5.29875^*^ | .27038 | .000 | -6.1512 | -4.4463 |
| *. The significance level of mean difference was 0.05. | | | | | | | |

**ZO-1**

| **ANOVA** | | | | | |
| --- | --- | --- | --- | --- | --- |
| value | | | | | |
|  | sum of squares | df | mean square | F | significance |
| interblock | 1.615 | 7 | .231 | 2.563 | .024 |
| intra-class | 4.861 | 54 | .090 |  |  |
| sum | 6.476 | 61 |  |  |  |

| **multiple comparisons** | | | | | | | |
| --- | --- | --- | --- | --- | --- | --- | --- |
| value | | | | | | | |
|  | (I) group | (J) group | Mean Difference  (I-J) | standard error | significance | 95% confidence interval | |
|  |  |  |  |  |  | lower limit | upper limit |
| Tukey HSD | 7.Model | 1.BD3150 | -.16375 | .15002 | .956 | -.6367 | .3092 |
|  |  | 2.BD400 | -.53125^*^ | .15002 | .018 | -1.0042 | -.0583 |
|  |  | 3.BD6256 | -.17375 | .15002 | .940 | -.6467 | .2992 |
|  |  | 4.BD5348 | -.19375 | .15002 | .898 | -.6667 | .2792 |
|  |  | 5.BB12 | .01375 | .15002 | 1.000 | -.4592 | .4867 |
|  |  | 6.MTX | -.10333 | .16204 | .998 | -.6142 | .4075 |
|  |  | 8.Control | -.24125 | .15002 | .743 | -.7142 | .2317 |
| *. The significance level of mean difference was 0.05. | | | | | | | |
